# Single-cell metabolomics profiling of somatosensory neurons in various stages of neuropathic pain

**DOI:** 10.1016/j.jbc.2025.108309

**Published:** 2025-02-13

**Authors:** Lin Yi, Tiepeng Liao, Man Yuan, Qi Chen, Wei Xiong, Hongying Zhu

**Affiliations:** 1 Hefei National Research Center for Physical Sciences at the Microscale, Division of Life Sciences and Medicine, University of Science and Technology of China, Hefei, China; 2 Anhui Province Key Laboratory of Biomedical Imaging and Intelligent Processing, Institute of Artificial Intelligence, Hefei Comprehensive National Science Center, Hefei, China; 3 CAS Key Laboratory of Brain Function and Disease, University of Science and Technology of China, Hefei, China; 4 Anhui Province Key Laboratory of Biomedical Aging Research, The First Affiliated Hospital of University of Science and Technology of China, Hefei, China

**Keywords:** neuropathic pain, single-cell mass spectrometry, glutamatergic neurons, metabolomics, primary somatosensory cortex

## Abstract

Metabolic alterations in the somatosensory cortex (S1) play a crucial role in neuropathic pain development, as evidenced by magnetic resonance spectroscopy and mass spectrometry analyses of brain homogenates. However, investigating metabolic changes in specific neuronal subtypes during neuropathic pain development remains challenging. Here, utilizing a recently developed technique called single-cell mass spectrometry (SCMS), we investigated metabolomic alterations within excitatory glutamatergic neurons located in the primary S1 during various stages of neuropathic pain. Specifically, we induced neuropathic pain in mice using a spared nerve injury (SNI) model and observed activation of glutamatergic neurons in layer II/III of S1 through c-Fos staining and electrophysiology. We profiled metabolic changes and performed pathway enrichment analysis in these neurons by single-cell mass spectrometry during both acute and subchronic phases of SNI. Further analyses revealed metabolites whose alterations significantly correlated with changes in pain thresholds, as well as distinct temporal patterns of metabolite expression during pain progression. From these analyses, we identified several key metabolites (homogentisic acid, phosphatidylcholine, phosphorylcholine, and rhein) and validated their causal roles in pain modulation *via* pharmacological interventions. Thus, our study provides a valuable resource for elucidating the neurometabolic regulatory mechanisms underlying neuropathic pain from a single-cell perspective.

Neuropathic pain is a prevalent disorder of the nervous system, typically resulting from damage to neural tissues (1, 2). Peripheral neuropathy or inflammatory injury induces alterations in cortical neural activity and cellular metabolism, leading to an exaggerated response to innocuous mechanical stimuli, known as mechanical allodynia (3, 4). Studies using functional MRI have revealed alterations in gray matter density and cortical thickness within the brains of patients suffering from chronic pain (5, 6). These brain regions—which include the somatosensory system (primary somatosensory cortex (S1), secondary somatosensory cortex, and insular cortex), limbic system (insular cortex and anterior cingulate cortex), and prefrontal cortex—constitute a parallel neural network consisting of multiple nociceptive signaling pathways (7). The primary S1 region receives input signals from the spinal–thalamic pathway (7, 8). Neuronal excitability in the S1 region is modulated by pain location, duration, and intensity (9, 10). Under conditions of chronic inflammation, activation of excitatory pyramidal neurons in the S1 contributes significantly to allodynia (11, 12), independent of depression-like states (13).

S1 is closely associated with pain (14, 15). Previous studies have primarily focused on the structural and functional plasticity changes of the S1 caused by pathological pain (12, 16), while its metabolic alterations have been predominantly detected through neuroimaging techniques such as PET and NMR (17, 18). A study based on magnetic resonance spectroscopy (MRS) revealed a decrease in the overall metabolite level, particularly N-acetyl-aspartate (NAA) in the left S1 and choline (Cho) in the right S1 of patients with low back pain (17, 18). However, our current knowledge regarding cortical neuronal metabolic alterations associated with allodynia remains limited.

Compelling evidence supports the pivotal role of the S1 pyramidal neurons in neuropathic pain (11, 12), yet further elucidation is required to comprehend how and to what extent the metabolism of these neurons is regulated during pain development. This is primarily due to the presence of diverse cell types in the S1, including excitatory and inhibitory neurons, astrocytes, microglia, among others (19, 20). Discerning the metabolic profiles of these distinct cell populations using mass spectrometry on tissue homogenates is an arduous task that poses a challenge for accurately characterizing cellular metabolic changes associated with neuropathic pain. Recently, in response to the dearth of precise intracellular metabolite measurement, we have developed a novel technique called single-cell mass spectrometry (SCMS), which combines patch-clamp and induced nanoelectrospray ionization-tandem mass spectrometry techniques (21, 22). This innovative approach empowers us to scrutinize the intracellular metabolome of individual neurons. In this study, we utilized this pioneering technique to analyze metabolomics changes within single excitatory neurons located in the S1 during various stages of neuropathic pain development.

## Results

### Somatosensory glutamatergic neurons are activated during SNI development

**Figure 1:**
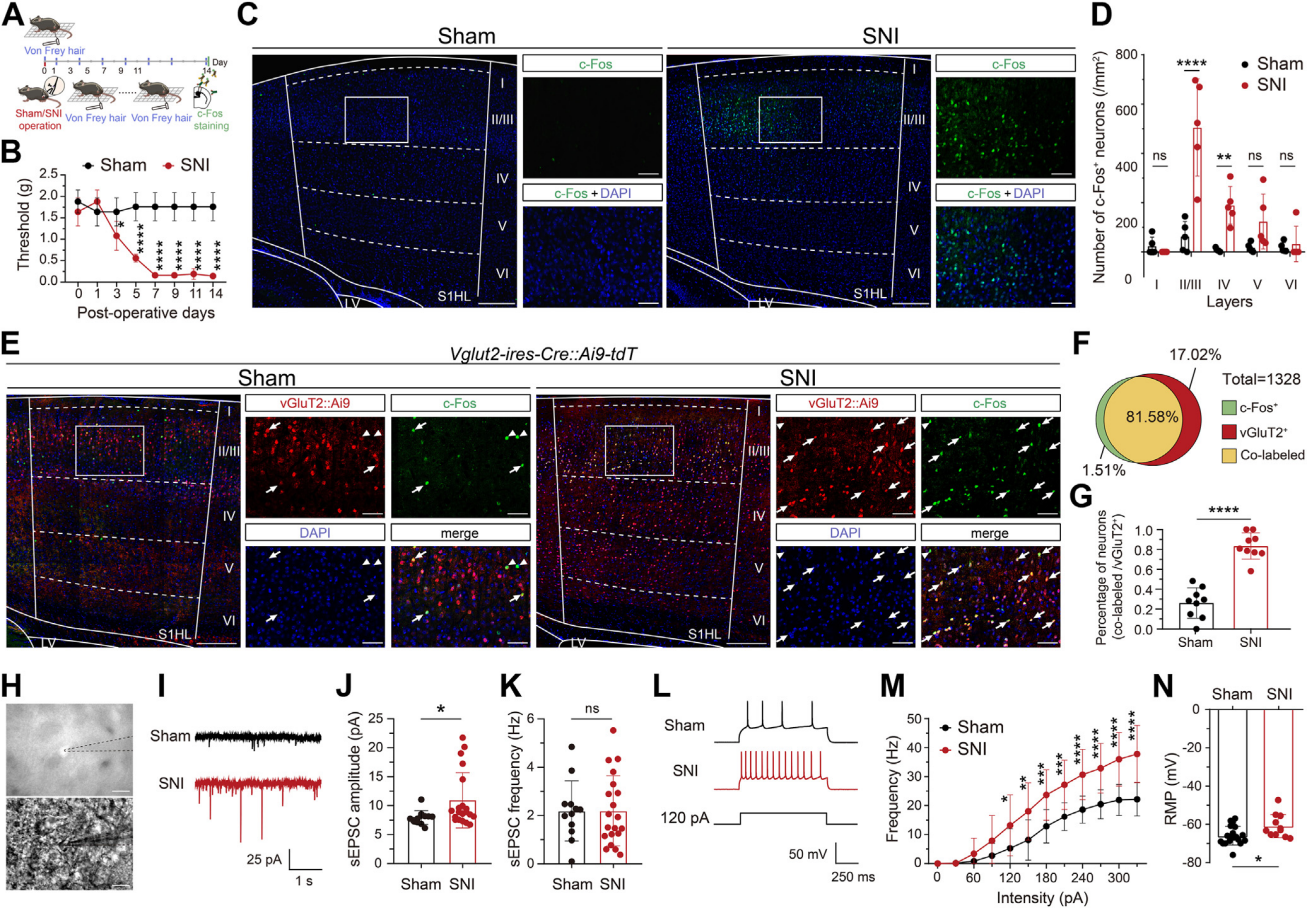
**Activities of glutamatergic neurons in S1**_**HL**_**layer II/III following SNI operations.***A*, schematic diagram of experimental schedule following sham/SNI operations. After baseline measurements, both groups of mice underwent surgical procedures. Subsequently, the von Frey Hair test was conducted on POD1, POD3, POD5, POD7, POD9, POD11, and POD14 prior to sacrificing the mice for c-Fos immunostaining. *B*, the mechanical threshold on the *left hind paw* assessed using the von Frey test throughout the postoperative time course revealed significant differences between the sham (n = 5) and SNI (n = 5) groups, as evidenced by a repeated measures two-way ANOVA (significant effects of time: F(2.797, 22.38) = 21.67, *p* < 0.0001; SNI treatment: F(1, 8) = 122.1, *p* < 0.0001); and their interaction: F(7, 56) = 23.43, *p* < 0.0001). *C*, representative images depicting c-Fos–positive neurons (labeled in *green*) across all layers in the S1_HL_ region of both sham-operated and SNI mice. The scale bar represents 200 μm and 50 μm (inset). *D*, the number of cells expressing c-Fos in the S1_HL_ region of sham and SNI mice in layers I, II/III, IV, V, and VI, expressed per mm^2^ area. Each data point in the graph represents c-Fos expression quantified from one brain slice, with five slices from three mice per group. Significant effects of layers: F(4, 40) = 17.57 = 21.67, *p* < 0.0001; SNI treatment: F(1, 40) = 38.06, *p* < 0.0001); and their interaction: F(4, 40) = 12.52, *p* < 0.0001. *E*, representative images showing c-Fos–positive neurons (*green*), vGluT2-positive neurons (*red*), and colabeled neurons (*yellow*) in S1_HL_ layer II/III of sham/SNI vGluT2-Cre::Ai9 mice. The scale bar represents 200 μm and 50 μm (inset). *F*, a pie chart representing the relative proportions of c-Fos–positive neurons, vGluT2-positive neurons, and colabeled neurons in S1_HL_ layer II/III of SNI mice. *G*, the proportion of colabeled neurons in c-Fos–positive neurons from sham (n = 3) and SNI mice (n = 3). Each data point in the graph represents the quantification of colabeling, c-Fos, and vGluT2 expression from one brain slice, with nine slices analyzed per group, derived from three mice. *H*, a DIC image (*top*) and a fluorescence image (*bottom*) of glutamatergic neurons in brain slices of vGluT2-Cre::Ai9 mice during patch-clamp recording. The scale bar represents 50 μm. *I*, representative traces of sEPSC of S1_HL_ layer II/III glutamatergic neurons from sham and SNI mice. *J* and *K*, quantified amplifies and frequencies of sEPSC of S1_HL_ layer II/III glutamatergic neurons from sham and SNI mice. *L*, representative action potential evoked by a 1-s long depolarizing current (120 pA) in S1_HL_ layer II/III glutamatergic neurons from sham and SNI mice. *M* and *N*, input-output curves and resting membrane potential of S1_HL_ layer II/III glutamatergic neurons from sham and SNI mice. Input-output curves was evidenced by two-way ANOVA (significant effects of intensity: F (11, 281) = 82.19, *p* < 0.0001; SNI treatment: F(1, 281) = 132.4, *p* < 0.0001); and their interaction: F(11, 281) = 4.512, *p* < 0.0001). Data are presented as mean ± SD. ∗*p* < 0.05, ∗∗*p* < 0.01, ∗∗∗*p* < 0.001, and ∗∗∗∗*p* < 0.0001 by unpaired *t* tests (for *panel G, K*, and *N*), Mann–Whitney *U* test (for *panel J*), two-way ANOVA with Bonferroni *post hoc* tests (for *panel D* and *M*) and repeated measures two-way ANOVA with Bonferroni's corrections (for *panel B*); ns, not significant (*p* > 0.05). DIC, differential interference contrast; POD, postoperative day; sEPSC, spontaneous excitatory postsynaptic current.

We have established a mouse model of spared nerve injury (SNI) to investigate the development of allodynia associated with tissue damage (23) (Fig. 1*A*). The SNI mice exhibited a significant and progressive decrease in their mechanical pain thresholds when compared to the sham group (Fig. 1*B*). The allodynia in SNI mice reached its zenith on postoperative day 7 (POD7) and persisted until POD14. Given that neuropathic pain leads to hyperexcitability of primary S1 (S1) (24), we conducted an immunofluorescence analysis of c-Fos expression to investigate neuron activity in contralateral S1 2 weeks postsurgery. A significantly increased expression of c-Fos was observed in the hind limb subregion of the contralateral S1 (S1_HL_), particularly in layer II/III, on POD14 in SNI mice compared to sham mice (Fig. 1, *C* and *D*). This suggests that neurons in the contralateral S1_HL_ were activated during the progression of neuropathic pain. The activated neuron subtype in layer II/III of the contralateral S1_HL_ exhibited a predominance of excitatory glutamatergic neurons, as confirmed by c-Fos staining in vGluT2-Cre::Ai9 mice with SNI on POD14 (Fig. 1, *E*–*G*), whose glutamatergic neurons have been labeled with tdTomato fluorescence protein (25).

We next performed whole-cell patch-clamp recordings to quantify the spontaneous excitatory postsynaptic current (sEPSC) and action potential (AP) of contralateral S1_HL_ glutamatergic neurons on brain slices obtained from vGluT2-Cre::Ai9 mice with or without SNI on POD14 (Fig. 1*H*). The amplitude of sEPSC is significantly elevated in SNI mice compared to sham mice, while the frequency of sEPSC remains unaltered (Fig. 1, *I*–*K*). Furthermore, we observed a noteworthy increase in action potential frequency and enhanced resting membrane potential (RMP) (Fig. 1, *L*–*N*). These findings collectively indicate that activation of glutamatergic neurons within the contralateral S1_HL_ layer II/III has occurred during SNI development.

### Single-cell metabolomic changes of somatosensory glutamatergic neurons in the acute phase of SNI

**Figure 2:**
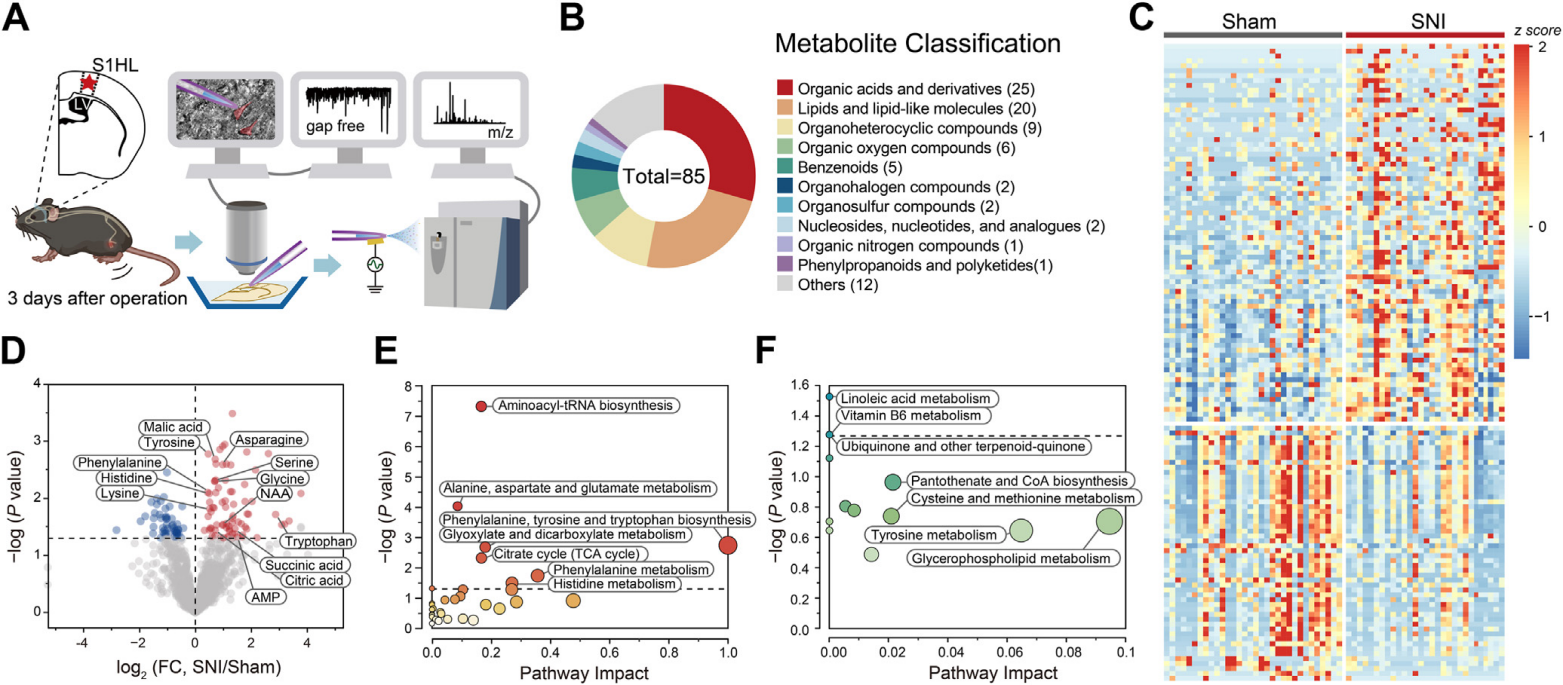
**Single-cell metabolomic changes in the glutamatergic neurons of S1**_**HL**_**layer II/III on POD3.***A*, schematic depicting the working flow of the SCMS experiment on the S1_HL_ layer II/III glutamatergic neurons on POD3. *B*, a pie chart depicting the superclassification of differentially abundant metabolites between sham and SNI mice during the acute phase of SNI. *C*, heat map of characteristic metabolites and their relative intensities in each neuron of sham and SNI mice. Color indicates z scores of metabolites in each sample. *D*, volcano plot showing correlations between *p* values (evaluated by two-sided Student's *t* tests) and relative intensities (FCs) for all detected metabolites in sham and SNI mice. *Red and blue dots* represent characteristic metabolites with a significant difference. *E* and *F*, MetaboAnalyst 5.0-based pathway analysis of the upregulated and downregulated metabolites in SNI group compared to sham group. *x-*axis represents the impact of the identified metabolites on the indicated pathway, while *y-*axis indicates the extent to which the designated pathway is enriched in the identified metabolites. Color intensity ranging from *white to red/blue* indicates the level of statistical significance, while the *size of circles* represents the impact of pathways. POD, postoperative day; SCMS, single-cell mass spectrometry

Previous research has revealed pathophysiological alterations in the central nervous system during the transition from the acute (POD3) to subchronic (POD14) phases of SNI (26, 27, 28), prompting us to focus on these two critical time points in neuropathic pain development. Specifically, we employed the SCMS technique to investigate metabolomic changes of single glutamertagic neurons on POD3 in the vGluT2-Cre::Ai9 mice with SNI (Fig. 2*A*). In the *m/z* range of 50 to 750, a total of 1205 ion signals were detected with occurrence frequency in all neurons exceeding 20% and with a signal-to-noise ratio >3. To ensure accuracy for subsequent analysis, the data of an individual cell was exclusive if least than 20% of naturally occurring amino acids were detected (Fig. S1*A*). After the above rigorous selection process, 1205 signals were deemed worthy of meeting the criteria. Among these, 550 metabolites—including lipids (n = 123), organic acids (n = 106), organic oxygen compounds (n = 76), and organoheterocyclic compounds (n = 58)—were further identified based on the Human Metabolome Database (HMDB) (Fig. S1*B*). For this study, metabolomic data were collected from two separate experiments, each utilizing three pairs of mice. In the acute phase of SNI (postoperative day 3), a total of 61 samples were obtained from two groups of glutamatergic neurons.

The metabolomics data at single-cell resolution revealed significant alterations in 85 metabolites within glutamatergic neurons located in layer II/III of the S1_HL_ region of mice subjected to SNI on POD3. These perturbed metabolites encompassed organic acids (n = 25), lipids (n = 20), organoheterocyclic compounds (n = 9), and organic oxygen compounds (n = 6) among others (Fig. 2*B*). According to the volcano plot and heat map, 57 metabolites exhibited increased levels while 28 metabolites displayed decreased levels (Fig. 2, *C* and *D* and Table S1). Notably, the signal intensities of metabolites including AMP, asparagine, guanine, phenylalanine, histidine, and tryptophan were found to be elevated in the SNI group of mice, which was accordance with previous investigations (29, 30, 31, 32). Pyridoxic acid was found decreased in SNI group, consistent with its role in pain management (33).

Metabolic pathway analysis of all significantly upregulated and downregulated metabolites, utilizing the Kyoto Encyclopedia of Genes and Genomes database, revealed nine significantly upregulated metabolic pathways (Fig. 2*E*). Notably, alterations in four of these pathways (“aminoacyl-tRNA biosynthesis,” “alanine, aspartate, and glutamate metabolism,” “phenylalanine, tyrosine, and tryptophan biosynthesis,” and “histidine metabolism”) mirrored those previously observed in serum and urine samples (34, 35). Table S2 provides a comprehensive overview of the results obtained from metabolic pathway analysis. “Aminoacyl-tRNA biosynthesis” emerged as the most significant upregulated pathway associated with acute phase of neuropathic pain, with nine amino acids (alanine, asparagine, glycine, histidine, lysine, phenylalanine, serine, tryptophan, and tyrosine) significantly increased in SNI mice's S1_HL_ layer II/III glutamatergic neurons on POD3. The second significantly upregulated pathway was the “alanine, aspartic acid, and glutamic acid metabolism.” N-Acetyl-aspartate, asparagine, alanine, citrate, and succinate were found to be significantly elevated in this pathway. Furthermore, increased levels of citrate and succinate also impacted the “citric acid cycle.” The pathway “phenylalanine, tyrosine, and tryptophan biosynthesis” impacted the most (impact score = 1), with a particular involvement of phenylalanine and tyrosine. Correspondingly, only the metabolic pathway of “linoleic acid metabolism” was significantly downregulated (Fig. 2*F*).

**Figure 3:**
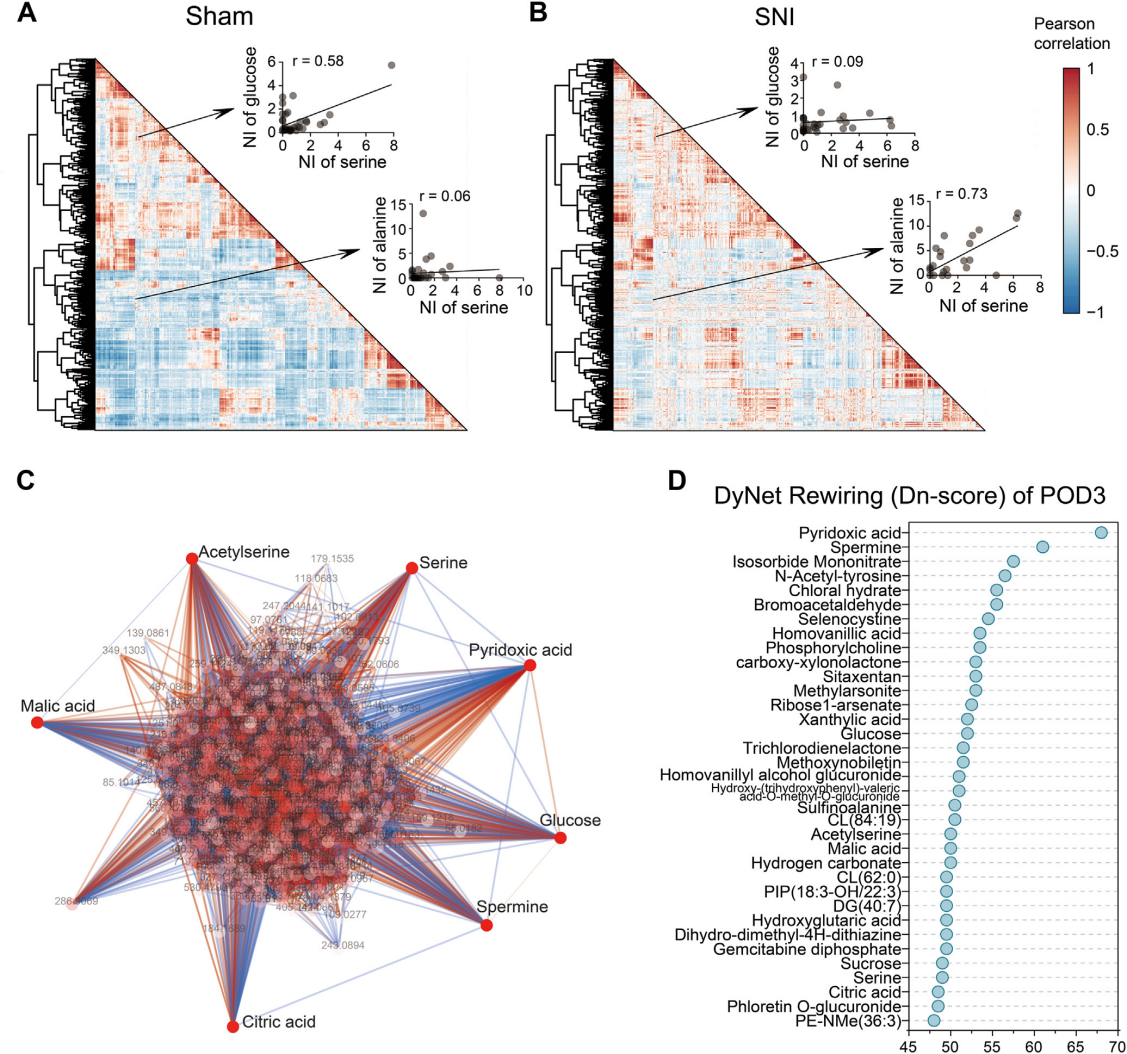
**Correlation between metabolites in the glutamatergic neurons of S1**_**HL**_**layer II/III during the acute phase of SNI.***A* and *B*, Pearson correlation coefficients were calculated among the all the metabolites recognized (n = 616), visualized by heat maps, in sham mice and in SNI mice, respectively. Pearson's correlation values span from −1 (*blue*, negative correlation) to 1 (*red*, positive correlation). The insets show the correlation between alanine-serine and glucose-serine as examples. *C*, a hub diagram illustrating a metabolite remodeling network was constructed by incorporating correlated metabolite pairs (|r| > 0.5) from the sham groups, along with additional metabolite pairs identified in the SNI group. The remodeling score of the metabolite it represents is positively correlated with the intensity of color in the knot. The *red line* indicates a stronger correlation between metabolite pairs in the SNI group, whereas the *blue line* indicates a stronger correlation between metabolite pairs in the sham group. *D*, a metabolite remodeling network was constructed by incorporating metabolite pairs with relatively high correlation (|r| > 0.5) from the sham groups, along with additional metabolite pairs identified in the SNI group. Metabolites ranked based on the remodeling score calculated using the DyNet algorithm. NI, normalized intensity.

Next, we conducted correlation analysis on the metabolites of sham and SNI group, respectively. The Pearson correlation coefficient between any two metabolites was calculated, as illustrated by the heat map (Fig. 3, *A* and *B*). Metabolite pairs with high correlations often reveals direct or indirect synergistic interactions (36, 37), and comparing the sham and SNI groups allows us to observe shifts in these correlations. For instance, the correlation between serine and its precursor metabolite alanine was significantly stronger in the SNI group than in the sham group. Conversely, the correlation between serine and another precursor metabolite, glucose (38), is diminished in the SNI group mice at POD3. These findings suggest that the upstream metabolism of serine is disturbed in the early stages of pain.

Furthermore, we constructed a metabolite remodeling network using metabolites with relatively high correlation (|r| > 0.5) in both the sham and SNI groups. Subsequently, we performed a comparative analysis of the intersection of the two sets of network components, by generating a hub diagram showcasing the meticulous reconstruction of metabolite correlations. By computing the remodeling fraction, we obtained changes in correlation between each metabolite and all other metabolites (Table S3). Metabolites were then ranked according to their remodeling scores calculated with the DyNet algorithm (Fig. 3*C*). In the top 40 metabolites with the highest remodeling scores, six were significantly upregulated during the acute phase of SNI, including citric acid and malic acid. Considering citric acid and malic acid were significantly upregulated in early stage (Fig. S4), and metabolic pathway analysis revealed that citric acid cycle, the pathways involving these two metabolites, were also altered in the SNI group of mice, suggesting that citric acid cycle may be affected in metabolic network during acte phase of pain. Notably, glucose were among the top 40 metabolites ranked by remodeling score (Fig. 3*D* and Table S3), which is consistent with the observations reported in previous studies (39, 40). Pyridoxic acid exhibited the highest degree of remodeling, suggesting potential alterations to vitamin B6 metabolism during SNI (41, 42).

### Single-cell metabolomics changes of somatosensory neurons in the subchronic phase of SNI

**Figure 4:**
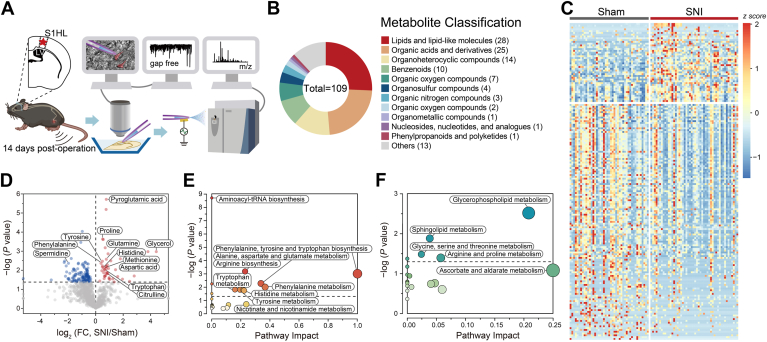
**Single-cell metabolomic changes in the glutamatergic neurons of S1**_**HL**_**layer II/III on POD14.***A*, schematic depicting the working flow of the SCMS experiment on the S1_HL_ layer II/III glutamatergic neurons on POD14. *B*, a pie chart depicting the superclassification of differentially abundant metabolites between sham and SNI mice during the subchronic phase of SNI. *C*, heat map of characteristic metabolites and their relative intensities in each neuron of sham and SNI mice. Color indicates z scores of metabolites in each sample. *D*, volcano plot showing correlations between *p* values (evaluated by two-sided Student's *t* tests) and relative intensities (FCs) for all detected metabolites in sham and SNI mice. *Red and blue dots* represent characteristic metabolites with a significant difference. *E* and *F*, MetaboAnalyst 5.0 pathway analysis based on KEGG database of the upregulated and downregulated metabolites in SNI group compared to sham group. *x-*axis represents the impact of the identified metabolites on the indicated pathway, while *y*-axis indicates the extent to which the designated pathway is enriched in the identified metabolites. Color intensity ranging from *white to red/blue* indicates the level of statistical significance, while the *size of circles* represents the impact of pathways. KEGG, Kyoto Encyclopedia of Genes and Genomes; POD, postoperative day; SCMS, single-cell mass spectrometry.

We persisted in exploring the alterations of single-cell metabolome in glutamatergic neurons situated in layer II/III of the S1_HL_ region from vGluT2-Cre::Ai9 mice on POD14 (Fig. 4*A*). Metabolites with significant changes on POD14 included lipids (n = 28), organic acids (n = 25), organoheterocyclic compounds (n = 14), and benzenoids (n = 10) (Fig. 4*B*).Our analysis has revealed that a remarkable shift in 109 metabolites occurred, with 34 being upregulated and 75 downregulated, between SNI mice and sham controls (Fig. 4, *C* and *D* and Table S4), suggesting a decreased metabolic activity of S1_HL_ glutamate neurons during the subchronic phase of SNI. This is in line with the findings of clinical magnetic resonance spectroscopy studies, which have shown that patients with chronic low back pain exhibit reduced concentrations of total metabolites in the S1 (17). The observed elevation of aspartic acid is consistent with the findings reported in serum samples from patients diagnosed with complex regional pain syndrome (29, 30).

For the metabolites exhibiting significant changes on POD14, a metabolic pathway analysis based on the Kyoto Encyclopedia of Genes and Genomes database was performed using MetaboAnalyst 5.0 (Fig. 4, *E* and *F* and Table S7). Similar with acute phase of SNI group, “aminoacyl-tRNA biosynthesis” remained the most significantly upregulated pathways during the subchronic phase of SNI, while “phenylalanine, tyrosine, and tryptophan biosynthesis” maintained its position as the most impactive pathway (impact score = 1). The second metabolic pathway that was significantly upregulated was “arginine biosynthesis,” which included citrulline, aspartic acid, and glutamine, which were also found to be significantly elevated. Next, we conducted metabolic pathway analysis on metabolites that exhibited significant reduction in the SNI group as compared to the sham group. Notably, “glycerophospholipid metabolism” pathway emerged as the top-ranked significantly downregulated pathway (Fig. 4*F*), owing to remarkable decreases in levels of several phosphatidylglycolamine, phosphatidylcholine (PtdCho), and phosphorylcholine (ChoP) in the SNI group on POD14 (Fig. S5). This finding aligns with prior research indicating a strong correlation between lipid metabolism and the development of chronic pain (43).

**Figure 5:**
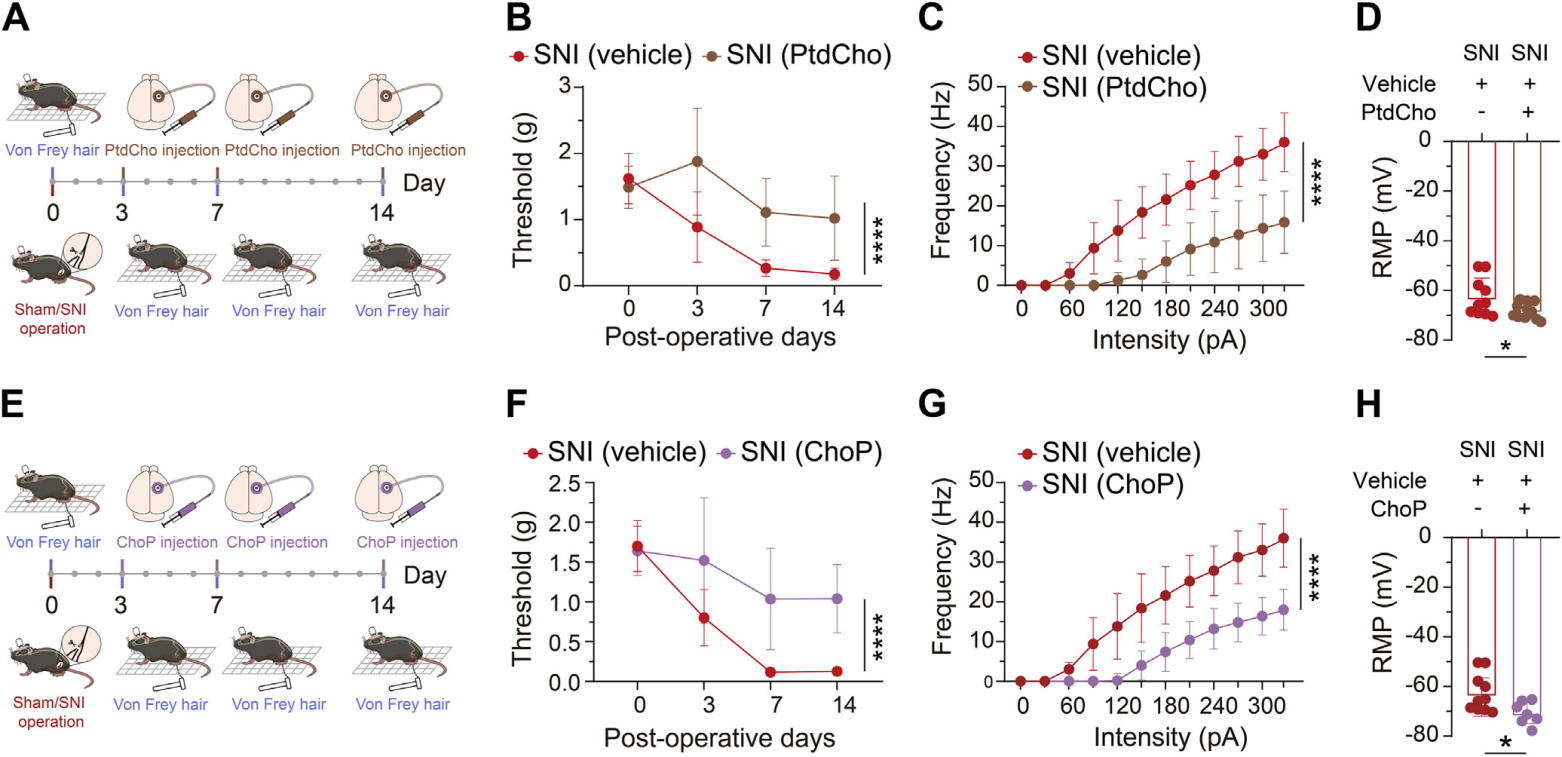
**Mechanical pain assessment and electrophysiological characterization following phosphatidylcholine and phosphorylcholine treatment in the SNI model.***A*, schematic diagram of the experimental procedure for assessing mechanical pain following the drug administration in vehicle-treated SNI group and phosphatidylcholine (PtdCho)-treated SNI group into the S1_HL_ layer II/III on POD3, POD7, and POD14 after SNI operation. *B*, mechanical pain thresholds measured 30 min after injection in vehicle-treated SNI group (n = 10) and PtdCho-treated SNI group (n = 10) on POD3, POD7, and POD14, with significant effects of time: F(2.106, 33.69) = 19.86, *p* < 0.0001; PtdCho treatment (1, 16): = 24.23, *p* < 0.001; and their interaction: F(3, 48) = 5.701, *p* < 0.01). *C* and *D*, input-output curves and resting membrane potential of S1_HL_ layer II/III glutamatergic neurons from vehicle-treated SNI group and PtdCho-treated SNI group. Input-output curves was evidenced by two-way ANOVA (significant effects of intensity: F (11, 192) = 45.75, *p* < 0.0001; PtdCho treatment: F(1, 192) = 218.6, *p* < 0.0001); and their interaction: F(11, 192) = 7.235, *p* < 0.0001). *E*, schematic diagram of the experimental procedure for assessing mechanical pain following the drug administration in vehicle-treated SNI group and phosphorylcholine (ChoP)-treated SNI group into the S1_HL_ layer II/III on POD3, POD7, and POD14 after SNI operation. *F*, mechanical pain thresholds measured 30 min after injection in vehicle-treated SNI group (n = 10) and ChoP-treated SNI group (n = 10) on POD3, POD7, and POD14, with significant effects of time: F(2.197, 35.16) = 23.43, *p* < 0.0001; ChoP treatment: F(1, 16) = 32.82, *p* < 0.0001; and their interaction: F(3, 48) = 4.591, *p* < 0.01). *G* and *H*, input-output curves and resting membrane potential of S1_HL_ layer II/III glutamatergic neurons from vehicle-treated SNI group and ChoP-treated SNI group. Input-output curves was evidenced by two-way ANOVA (significant effects of intensity: F(11, 192) = 69.26, *p* < 0.0001; ChoP treatment: F(1, 192) = 213.3, *p* < 0.0001); and their interaction: F(11, 192) = 7.128, *p* < 0.0001). Data are presented as mean ± SD. ∗*p* < 0.05, ∗∗*p* < 0.01, ∗∗∗*p* < 0.001, ∗∗∗∗*p* < 0.0001 by repeated measures two-way ANOVA with Bonferroni's corrections (for *panel B* and *F*), two-way ANOVA with Bonferroni *post hoc* tests (for *panel C* and *G*) and unpaired *t* tests (for *panel D* and *H*). POD, postoperative day.

Interestingly, we also observed that the “glycerophospholipid metabolism” pathway was downregulated on POD3 and POD14 (Fig. S5). To investigate whether this pathway contributes to the pain behaviors, we administered microinjections of PtdCho or ChoP into the S1_HL_ layer II/III of SNI mice on POD3, POD7, and POD14 to replenish the levels of these compounds. Both PtdCho (5 μg per mouse) and ChoP (1 μg per mouse) resulted in a significant increase in the mechanical pain threshold (Fig. 5, *A*, *B*, *E*, and *F*).

To further investigate whether “glycerophospholipid metabolism” pathway contributes to neuronal hyperactivity in layer II/III of the S1_HL_, we conducted electrophysiological recordings of action potentials following incubation of PtdCho or choline phosphate (ChoP). The input-output curves and resting membrane potential data revealed a significant reduction in neuronal excitability after incubation with PtdCho and ChoP (Fig. 5, *C*, *D*, *G*, and *H*). These findings indicate that supplementation with “glycerophospholipid metabolism” (PtdCho or ChoP) not only exerts analgesic effects but also normalizes hyperexcitability in neurons within the S1_HL_. This suggests that targeting glycerophospholipid metabolism may hold therapeutic potential for managing neuropathic pain.

**Figure 6:**
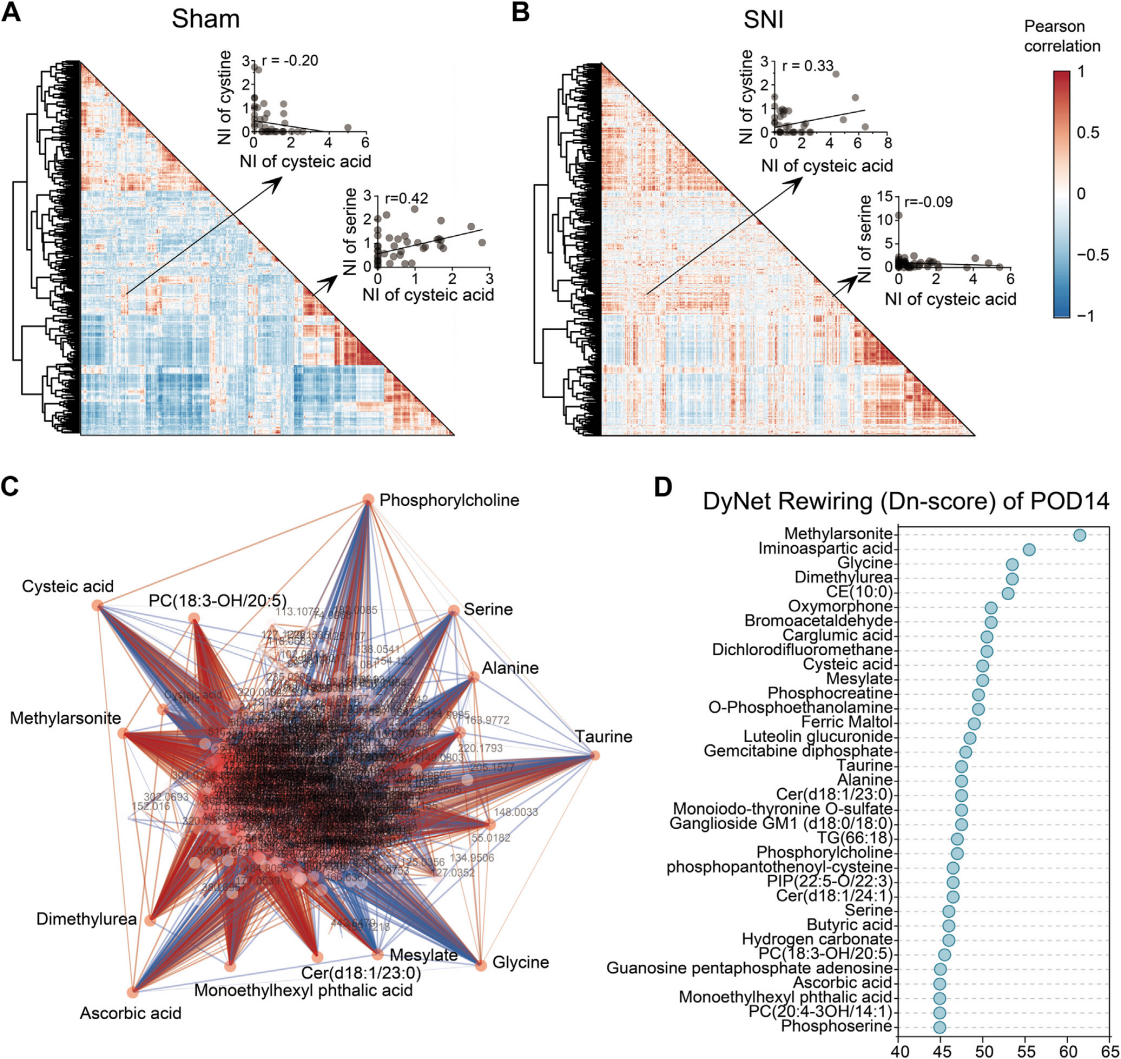
**Correlation between metabolites in the glutamatergic neurons of S1**_**HL**_**layer II/III during the sub-chronic phase of SNI.***A* and *B*, Pearson correlation coefficients were calculated among all the metabolites recognized (n = 616), visualized by heat maps, in sham mice and in SNI mice, respectively. Pearson's correlation values span from −1 (*blue*, negative correlation) to 1 (*red*, positive correlation). The insets show the correlation between cystine-cysteic acid and serine-cysteic acid as examples. *C*, a hub diagram illustrating a metabolite remodeling network was constructed by incorporating correlated metabolite pairs (|r| > 0.5) from the sham groups, along with additional metabolite pairs identified in the SNI group. The remodeling score of the metabolite it represents is positively correlated with the intensity of color in the knot. The *red line* indicates a stronger correlation between metabolite pairs in the SNI group, whereas the *blue line* indicates a stronger correlation between metabolite pairs in the sham group. *D*, a metabolite remodeling network was constructed by incorporating metabolite pairs with relatively high correlation (|r| > 0.5) from the sham groups, along with additional metabolite pairs identified in the SNI group. Metabolites ranked based on the remodeling score calculated using the DyNet algorithm. NI, normalized intensity.

Next, we generated correlation matrices of all metabolites (n = 616) for both the sham and SNI groups on POD14. The arrangement of metabolites in each group was based on hierarchical clustering order derived from Pearson correlation–based distance in the sham group (Fig. 6, *A* and *B* and Table S6). The pairwise correlation analysis illustrated significant alterations in the interplay between metabolites in the subchronic phase of SNI, as compared to the sham controls. As demonstrated by a representative example, there was a significantly higher positive correlation between cysteate and cystine in SNI mice than sham mice, while the correlation between cysteate and serine was reduced. Given that cysteate and cystine are both oxidative products of cysteine through different pathways (44, 45), these altered correlations suggest that neuropathic pain might modulate the oxidative stress status in the glutamatergic neurons in layer II/III of the S1_HL_.

Changes in the correlation between each metabolite and all others were obtained by computing the remodeling scores, which were calculated using the DyNet algorithm (Fig. 6*C*). Interestingly, seven metabolites that were significantly downregulated in the subchronic phase of pain appeared in the rewiring score 35 ranking list, along with the previously mentioned ChoP (Fig. 6*D*). Moreover, previously mentioned cysteate (cysteic acid) and serine were also found in the list. Furthermore, taurine exhibited a high rewiring score. This observation aligned with studies demonstrating elevated urinary taurine levels in patients with neuropathic pain (46, 47), suggest a potential biomarker role for taurine in neuropathic pain.

### Comparison of single-cell metabolome of somatosensory neurons between acute and subchronic SNI

**Figure 7:**
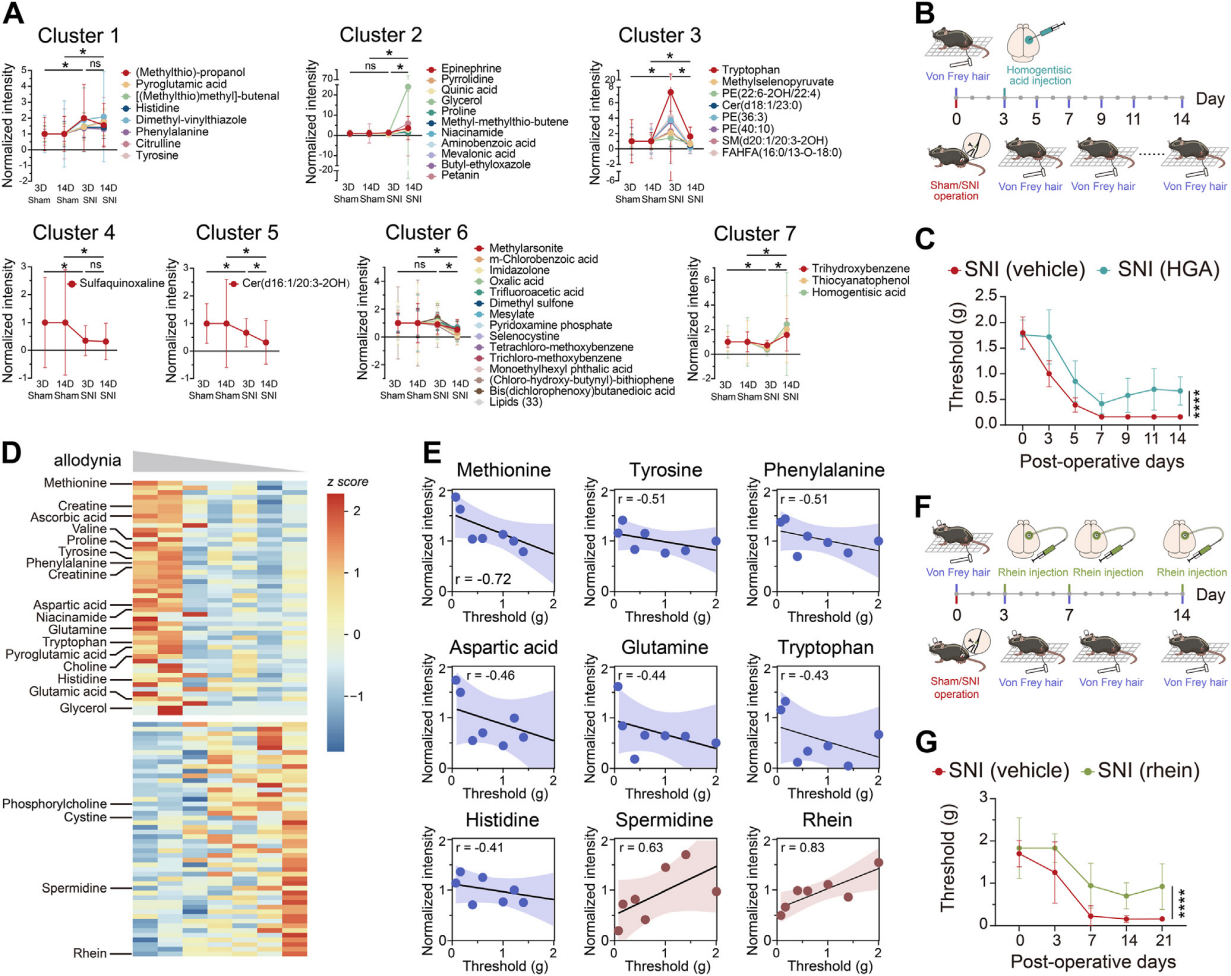
**Metabolic changes in different stages of neuropathic pain.***A*, distinct clusters of metabolites exhibiting divergent temporal changes observed in the glutamatergic neurons of layer II/III in the S1_HL_ region between mice subjected to SNI and sham surgery, at POD3 and POD14. *B*, schematic diagram of the experimental procedure for continuously assessing mechanical pain thresholds over a 14-day period following the injection of homogentisic acid (HGA) into the S1_HL_ layer II/III on postoperative day three (POD3). *C*, mechanical threshold of vehicle-treated SNI group (n = 6) and HGA-treated SNI group (n = 6) assessed 30 min after drug administration into the S1_HL_ layer II/III on POD3 and following 11 days, with significant effects of time: F(3.648, 36.48) = 52.68, *p* < 0.0001; HGA treatment: F(1, 10) = 41.96, *p* < 0.0001; and their interaction: F(6, 60) = 2.279, *p* < 0.1. *D*, heat map showing metabolites that downregulated or upregulated with decreased mechanical pain threshold. *E*, linear correlation between the average single-cell intensities of representative metabolites in glutamatergic neurons located in layer II/III of the S1_HL_ region in SNI mice and their mechanical pain threshold. *F*, schematic diagram of the experimental procedure for assessing mechanical pain following the drug administration in vehicle-treated SNI group and rhein-treated SNI group into the S1_HL_ layer II/III on POD3, POD7, POD14, and POD21 after SNI operation. *G*, mechanical pain thresholds measured 30 min after injection in vehicle-treated SNI group (n = 10) and rhein-treated SNI group (n = 12) on POD3, POD7, POD14, and POD21, with significant effects of time: F(2.755, 68.87) = 65.56, *p* < 0.0001; rhein treatment: F(1, 25) = 31.16, *p* < 0.0001; and their interaction: F(4, 100) = 2.577, *p* < 0.1. Data are presented as mean ± SD. ∗∗∗∗*p* < 0.0001 by repeated measures two-way ANOVA with Bonferroni's corrections (for *panel C* and *G*). HGA, homogentisic acid.

We conducted a comprehensive comparison and analysis of the metabolomics data obtained on POD3 and POD14 (Fig. S6). To further investigate the metabolic changes in glutamatergic neurons within S1_HL_ layer II/III on these two postoperative days, we normalized all data to their respective sham groups for comparative analysis. We then classified the metabolites into several clusters based on their significance and change trends between groups (Fig. 7*A*). Of the 21 metabolites that exhibited significant upregulation/downregulation during both acute and subchronic phases of SNI, eight metabolites displayed an increase during the acute phase and remained stable throughout the subchronic phase (cluster 1), 11 metabolites increased exclusively in the subchronic phase of SNI (cluster 2), eight metabolites increased during the acute phase but decreased in subchronic phase of SNI (cluster 3), 1 metabolite decreased during the acute phase but remained stable throughout the subchronic phase (cluster 4), 1 metabolite demonstrated a decrease during both stages (cluster 5), 47 metabolites decreased exclusively in the subchronic phase of SNI (cluster 6), and three metabolites decreased during the acute phase of SNI, followed by an increase in the subchronic phase (cluster 7) (Table S7). Therefore, it is hypothesized that metabolites in cluster 1 and cluster 4 may be closely associated with the acute phase of neuropathic pain, while those in cluster 2 and cluster 6 may be more closely linked to the subchronic phase. Metabolites involved in the transition from acute to subchronic phases of neuropathic pain are likely distributed across clusters three and 7. In cluster 5, ceramide exhibited a consistent reduction in both acute and subchronic phases of SNI, akin to the sphingomyelin ceramide metabolism disorder previously reported in the dorsal horn of rats with neuropathic pain (48).

Notably, we observed a significant decrease in homogentisic acid (HGA) on POD3, but not on POD14. To explore whether the metabolic changes occurring on POD3 could have a causal impact on the subsequent development of chronic pain, we microinjected HGA into the S1_HL_ layer II/III of SNI mice on POD3 and monitored their mechanical pain threshold over the following 11 days (Fig. 7*B*). The HGA treatment not only induced a substantial increase in the mechanical pain threshold in the SNI group on POD3 but also mitigated the subsequent development of allodynia following POD3 (Fig. 7*C*). This finding suggests that metabolic alterations during the early stages may have a causal relationship with the later stages of chronic pain development.

To identify representative metabolites associated with pain sensitivity, we conducted a correlation analysis between the levels of each metabolite in glutamatergic neurons within layer II/III of S1_HL_ and the mechanical pain threshold in mice. The top 50 metabolites with positive and negative correlations were visualized by heat map (Fig. 7*D* and Table S8). Metabolites that exhibited a positive correlation with the mechanical pain threshold in mice included rhein, spermidine and ChoP, all of which were downregulated following SNI.

Considering the robust positive correlation between rhein levels and pain threshold, we hypothesized that rhein supplementation might modulate somatosensory neuron activity, thereby exerting analgesic effects. To test this hypothesis, we administered rhein *via* microinjection into the S1_HL_ region of SNI mice on postoperative days 3, 7, 14, and 21. Mechanical pain thresholds were assessed 30 min following each injection. Our results demonstrated a significant increase in mechanical pain threshold in the rhein-treated SNI mice (Fig. 7, *F* and *G*). This finding is consistent with previous studies indicating that rhein and its analog diacerein possess analgesic properties in inflammatory pain models (49, 50).

In addition to rhein, spermidine has demonstrated the ability to activate voltage-dependent modulate transient receptor potential vanilloid channels (51) and reduce chronic constriction injury-induced neuropathic pain in rats (52). Several metabolites—including ascorbic acid, aspartic acid, creatinine, glutamine, histidine, methionine, phenylalanine, proline, pyroglutamic acid, tryptophan, and tyrosine—showed a negative correlation with the mechanical pain threshold in mice (Fig. 7*E*). Significantly, levels of histidine, phenylalanine, pyroglutamic acid, tryptophan, and tyrosine are elevated during both acute and subchronic phases of SNI in mice. This observation is consistent with previous studies conducted on serum, muscle, and brain tissue (29, 30, 53). The aromatic amino acids—tyrosine, phenylalanine, and tryptophan, previously highlighted for their interconnected metabolic pathways (34, 35, 53)—may have multifaceted roles in pain nociception mechanisms, encompassing the biosynthesis of catecholamines, serotonin, and nitric oxide production (54).

## Discussion

This study utilized SCMS technology (21, 22) to explore the metabolic alterations of a specific subtype of neurons in the brain during neuropathic pain. Prior investigations on metabolic changes during chronic pain predominantly relied upon conventional techniques such as GC/MS, LC/MS, and NMR, along with invasive (blood, synovial fluid, and cerebrospinal fluid) (55, 56, 57) and noninvasive (saliva and urine) (58, 59) sample detection utilizing chromatography or mass spectrometry. However, these methods are limited in their ability to resolve cell types due to the presence of various cells in blood and tissue homogenates, making it challenging to identify the specific contribution of a particular cell type to metabolite signals. In contrast, our approach offers significant advantages and innovations compared to traditional studies that utilize homogenized brain tissue without considering cellular heterogeneity.

The implementation of SCMS technology empowers us to discern the metabolic alterations of individual cells, thereby facilitating heightened precision and enhanced accuracy. Specifically, our focus was directed toward the S1 region of the brain, a complex network comprising various cell types such as excitatory neurons, inhibitory neurons, astrocytes, and microglia. Our findings revealed that during neuropathic pain, there was an increase in excitation among excitatory neurons located in S1 layer II/III. This underscores the significance of studying specific metabolic changes within this particular group of cells. It was revealed that the amino acids exhibited the highest prevalence among the metabolites that displayed a significant shift between the sham and SNI group. To be specific, six amino acids (tyrosine, phenylalanine, alanine, histidine, citrulline, and tryptophan) exhibited a considerable surge during both acute to subchronic phases of neuropathic pain progression. The observed regulation of amino acids aligns with previous research findings that have reported altered levels of amino acids in blood, synovial fluid, or cerebrospinal fluid of patients suffering from various types of pain such as migraine (60), osteoarthritis (31, 56), fibromyalgia (61), musculoskeletal pain (62), and complex regional pain syndrome (63). The heightened levels of aspartic acid have also been detected in serum samples from patients diagnosed with complex regional pain syndrome, potentially serving as an excitatory neurotransmitter that contributes to the activation of N-methyl-D-aspartate receptors and the onset of persistent nociceptive responses (allodynia) associated with chronic pain (64). Another significant metabolite that demonstrates an increase in the subchronic phase of neuropathic pain, as revealed by this study, is lactic acid. This compound is associated with a metabolic shift that has been linked to various painful conditions (47, 65). Lactic acid, the primary product of glycolysis, acts as a signaling molecule for neuronal–glia interactions, as well as neuronal plasticity (66, 67), implying potential impairment of glycolytic function in somatosensory neurons during neuralgia.

Relevant modifications in the correlations between metabolites imply a metabolic reconfiguration within excitatory neurons located in S1 layer II/III during neuropathic pain. There are multiple reasons for the occurrence of such metabolic remodeling within somatosensory neurons in neuropathic pain. First, such remodeling can be attributed to genomic or proteomic changes in these neurons. Previous studies have identified alterations in transcription and protein modifications of enzymes involved in cellular metabolism (68). Additionally, alterations have been noted in the transporters accountable for specific small molecular metabolites (69, 70). These modifications are likely to give rise to changes in the concentrations of intracellular metabolites, thereby leading to a comprehensive restructuring of the metabolic network. Second, other behavioral changes during the progression of neuropathic pain may also contribute to the metabolic remodeling in somatosensory neurons. For instance, neuropathic pain may lead to alterations in dietary habits (71) and sleep patterns (72), both of exert a profound influence on organismal metabolism. The remodeling of the metabolic network in somatosensory neurons is not merely a consequence of long-term neural damage, but rather an indispensable contributing factor to neuropathic pain. For example, this study has revealed a plethora of modified metabolites, including an array of neurotransmitters and neuromodulators. Alterations in their levels possess the potential to disrupt neuronal signaling, thereby culminating in aberrant activation of neural circuits and the initiation of chronic pain. Furthermore, certain modified metabolites unveiled in this study, such as citrulline and lactic acid, have been previously reported to exhibit a strong correlation with synaptic plasticity (67, 73). Hence, modifications in these metabolites possess the potential to elicit enduring transformations in neuronal membrane structure and synaptic function, thereby instigating the onset of chronic pain.

## Experimental procedures

### Mice

All animal experiments were approved by the Institutional Animal Care and Use Committee of the University of Science and Technology of China (USTC) (Protocol No. USTCACUC26100124003) and conducted in accordance with the guidelines of the USTC and the Chinese Academy of Sciences. The mice were housed under specific pathogen-free conditions, with free access to food and water *ad libitum*, at a temperature range between 21 and 25 °C, following a 12/12 h light/dark cycle (lights off at 7 PM). Housing enrichment included nesting material, tunnels, and chew toys to promote natural behaviors and enhance environmental complexity. Mice were group housed to allow social interaction. C57BL/6J mice were purchased from Beijing Vital River Laboratory or Jackson Laboratories. Vglut2-ires-Cre (Strain #: 016963, referred to as vGluT2-Cre), Ai9 (Strain #: 007909) mice were originally obtained from Jackson Laboratories (25) and subsequently crossbred in our facility to generate vGluT2-Cre::Ai9 mice for immunoassay studies and sampling. Male mice, aged 6 to 8 weeks, were used for all experiments. Experimenters remained blinded to all experimental conditions until data collection was completed. Unless otherwise specified, the control and treatment groups in all experiments were randomly assigned.

### Spared nerve injury

Male mice at the age of 6 to 8 weeks were anesthetized with 0.5% sodium pentobarbital at a dose of 100 mg/kg (74). The SNI procedure involved a 1 cm incision in the longitudinal direction proximal to the knee, followed by blunt dissection to expose the sciatic nerve terminal branches. Tight ligation of the tibial and common peroneal branches of the sciatic nerve was performed with a 6-0 silk suture, followed by distal transection while preserving the sural nerve integrity. During ligation, limb withdrawal was observed as an indicator of nerve engagement (23). The muscle layer was then sutured, followed by the skin closure, and animals were returned to their cages. In sham-operated controls, only exposure of the sciatic nerve terminal branches was performed without ligation. The wounds were subsequently closed and animals returned to their cages.

### Measures of mechanical allodynia

A time course of tactile sensitivity was assessed in C57BL/6J and vGluT2-Cre::Ai9 mice presurgery and postsurgery. The animals were habituated for 2 h in test compartments placed on an elevated mesh-bottomed platform to access the ventral hind paws. Each group of mice was tested every other day prior to and following SNI to prevent overstimulation or habituation during testing. Both groups of mice were utilized to establish baseline, followed by assessments at 1, 3, 5, 7, 9, 11, and 14 days after operation.

Mechanical allodynia was evaluated by applying von Frey filaments with a range of 0.07 to 2 g onto the sural territory of the hind paw plantar surface using an up-down method for a duration of 2 to 3 s. The evaluation commenced with a 0.6 g von Frey filament and subsequently, as allodynia progressed, a weaker stimulus was employed at the onset. In each subsequent trial, if the filament elicited no response, a stronger stimulus was selected. A positive response to the filament was defined as immediate licking/biting, flinching, or fast withdrawal of the stimulated paw observed in more than half of the cases.

As mice undergoing SNI develop exquisite hypersensitivity during the first week following nerve injury, and this study relies on precise determination of its onset, we took great care to avoid introducing any confounding factors when evaluating sensitivity. To measure tactile allodynia, we also took special precautions to minimize the impact of repeated measures resulting from repetitive von Frey stimulation by allowing at least 30 s between each stimulation.

### Immunofluorescence

Ninety minutes after von Frey Hair behavioral tests, the mice were anesthetized with 0.5% sodium pentobarbital and transcardially perfused with 20 ml PBS followed by 20 ml of 4% (w/v) paraformaldehyde. The brains were then removed and postfixed in 4% paraformaldehyde at 4 °C overnight, followed by dehydration in a solution of 30% (w/v) sucrose in PBS at 4 °C overnight. Subsequently, the brain was embedded in optimum cutting temperature compound and sectioned at a thickness of 40 μm using a cryostat (CM 1860, LEICA Microsystems) at −20 °C. Prior to this, blocking was performed for 1 h in PBS containing 5% (w/v) goat serum and 0.3% (w/v) Triton X-100. The sections were then incubated overnight at 4 °C with primary antibody against c-Fos (1:1000, Abcam, ab190289). Following three 5-min washes with PBS, the sections were incubated in goat anti-rabbit (1:1000, A-11034, Invitrogen) for 2 h. After another three 5-min washes with PBS, the sections were stained with 4′,6-diamidino-2-phenylindole (D9542, Sigma-Aldrich) for 10 min. The brain sections were then washed with PBS and mounted on gelatin-coated slides before being air-dried and cover-slipped using an 80% (w/v) glycerol solution.

Immunofluorescence images were captured using a Zeiss LSM 880 confocal microscope with 10× and 20× objective lens, producing images of 1024 × 1024 pixels. To capture extensive regions for an overview of the slices, we employed the tile scan function, stitching images together with a 10% overlap. The quantification of c-Fos–positive neurons was performed using ImageJ software (National Institutes of Health, https://imagej.nih.gov/ij/), for three brain slices per mouse, with measurements taken from five random fields-of-view on each slice.

### Brain slice preparation

The primary S1 slices were obtained from vGluT2-Cre::Ai9 mice aged P56-P70. The mice were euthanized and their brains were quickly extracted from the skull, then immersed in ice-cold preoxygenated cutting solution containing sucrose 194 mM, NaCl 30 mM, NaHCO_3_ 26 mM, MgCl_2_ 1 mM, glucose 10 mM, KCl 4.5 mM, and NaH_2_PO_4_ 1.2 mM at pH level of 7.4. Coronal sections (300 μm) were sliced using a Vibratome (VT 1200S, Leica). The tissue slices were incubated in a holding chamber filled with oxygenated artificial cerebrospinal fluid (aCSF) containing 124 mM NaCl, 26 mM NaHCO_3_, 4.5 mM KCl, 1.2 mM NaH_2_PO_4_, 1 mM MgCl_2_, 2 mM CaCl_2_, and 10 mM glucose (pH = 7.4; osmolality = 315 mOsm/kg), supplemented with a gas mixture of 95% O_2_ and 5% CO_2_. Mouse brain slices (in coronal or sagittal planes) that had suffered mechanical injury were incubated in oxygenated aCSF at 32 °C for approximately 30 min, followed by cooling to room temperature (21–23 °C) for at least another 30 min prior to recording.

### Electrophysiological recording

Slices were continuously transferred into the recording chamber and perfused with aCSF at a rate of 3 to 4 ml/min, while being maintained at 28 °C. Neurons were visualized using a fixed-stage microscope (BX50WI, Olympus) equipped with differential interference contrast and infrared illumination. For sEPSC and action potential experiments, brain slices from the contralateral hemisphere S1_HL_ area of Vglut2-Cre:: Ai9 mice were utilized. Only glutamatergic neurons expressing tdTomato protein were selected for testing. We conducted all recordings in whole-cell voltage-clamp mode using borosilicate glass pipettes (5–7 MΩ) filled with internal solutions. The pH 7.2 internal solution for recording excitatory postsynaptic currents and action potentials contained K-gluconate 145 mM, Hepes 5 mM, Mg-ATP 5 mM, Na-GTP 0.2 mM, and EGTA 10 mM. The sEPSCs were recorded at a holding potential of −70 mV and bicuculline (20 μM) was added to the aCSF to block inhibitory neurotransmitter receptors (GABA_A_ and glycine receptors). Experimental results were excluded if the series resistance varied by more than 15% or exceeded 25 MΩ.

### Stereotaxic surgery and drug administration

Six-to-eight-week-old male C57BL/6J mice were used for the stereotaxic implantation of injection cannulas targeting the right primary S1 (S1_HL_ layer II/III). Mice were anesthetized with 0.5% sodium pentobarbital at a dose of 100 mg/kg and secured in a stereotaxic apparatus. Following a midline scalp incision, a small craniotomy was performed above the target site using a hand-held drill. The stereotaxic coordinates for cannula placement, relative to bregma, were anteroposterior: −1.4 mm, mediolateral: −0.5 mm, and dorsoventral: −0.8 mm. A stainless-steel guide cannula (internal diameter: 0.25 mm) was inserted into the right hemisphere only and secured with dental cement. To prevent occlusion, dummy cannulas were inserted into the guides. Mice were returned to their home cages and allowed to recover for 7 days before undergoing spared nerve injury surgery. On drug administration days, 1 μl of vehicle or drug solution was slowly injected through the cannula, which was left in place for 5 min before being removed. Mechanical pain thresholds were then measured 30 min postinjection.

The injection solutions were prepared as follows: rhein (#: R7269, Sigma) (1 μg/μl, dissolved in 5% dimethyl sulfoxide), HGA (#: H0751, Sigma) (250 μg/μl, dissolved in saline), PtdCho (#: 1535755, Sigma) (5 μg/μl, dissolved in dimethyl sulfoxide), and ChoP(#: P302169, Aladdin) (1 μg/μl, dissolved in saline). All solutions were adjusted to a physiological pH of approximately 7.0 prior before injection. Each solution was freshly prepared on the day of administration to ensure stability and accuracy.

### Single-neuron MS

Electrophysiological recordings and sampling were conducted on vGluT2-Cre::Ai9 mice, in which glutamatergic neurons were fluorescently labeled. Following extraction of the cell cytoplasmic chemical constituents, a capillary was connected to the single-neuron MS that had been previously described (46). The brain slices were transferred to recording chambers after recovering from mechanical injury. The neurons were selected at random for subsequent electrophysiological recording and MS analysis. We utilized a micromanipulator (MP-285, Sutter) to approach the neuron and patched it with borosilicate glass pipettes filled with pipette solution (NH_4_HCO_3_ 185 mM, and NH_4_Cl 80 mM) by applying negative pressure. The neurons were clamped at −70 mV after the patched cell membrane was ruptured by rapidly applied negative pressure. Following electrophysiological recording, negative pressure was applied to obtain cytoplasmic chemical constituents from the assayed neuron. Only neurons with seals >1 GΩ and nonruptured membranes were selected for analysis to avoid intracellular fluid dilution by aCSF. Once sufficient fluid was withdrawn from the cell, the patch pipette was quickly removed from the slice and then analyzed *via* MS.

An alternating current voltage of 4 kV amplitude at a frequency of approximately 500 Hz was applied externally to the spray capillary micropipette, while maintaining a distance of approximately 5 mm between the tip of the spray micropipette and the orifice of the MS instrument. High-resolution mass measurements were analyzed using an Exactive Plus MS instrument (Thermo Fisher Scientific). The primary experimental parameters for the instrument were established as follows: capillary temperature of 275 °C, S-lens radio frequency level of 50%, mass resolution of 70,000, maximum injection time of 10 ms, and microscan set to 1. Additional MS experiments were conducted using a Thermo Fisher Scientific LTQ Velos Pro MS instrument with the following parameters: capillary temperature at 275 °C, S-lens radio frequency level at 42%, maximum injection time set to 300 ms, and microscan also set to 1. The chemical structure was confirmed *via* tandem MS analysis using the LTQ Velos Pro instrument, which utilizes collision-induced dissociation with helium (He) as a background gas at 28% energy. Prior to our experiments, the commercial electrospray ionization source was removed. Our experiments were conducted in positive ion mode.

### Data preprocessing

Mass spectral data from each cell were stored in separate files and converted into mzXML format using ProteoWizard software (http://www.proteowizard.org/). The original data was preprocessed with XCMS, which included quality control, peak detection, filtering, and correspondence. The parameters of the XCMS program were consistent for all mice. Only cells that met the quality control criteria were selected for analysis. Only cells containing a minimum of 20% naturally occurring amino acids ions were selected for further analysis. Reliable metabolite signals were identified based on an SNR greater than three and a frequency exceeding 20% in all cells (Fig. S1*A*). After data processing, matrices were generated with row names representing *m/z* values and column names indicating sample names. Metabolites were identified by matching their accurate masses with information from the HMDB (http://www.hmdb.ca/). The margin of error was set to be less than 15 ppm. Some metabolites were further identified by comparing tandem mass spectrometry spectra obtained from neurons with data from the standards (Fig. S2) or the HMDB.

After checking the batch effect between the three pairs of mice using principal component analysis (Fig. S3), metabolites with a significance level of *p* < 0.05 were identified as characteristic metabolites. The z-score values of all matched metabolites were utilized to generate heat maps, where the z-score represents the deviation from the mean value of a group of values and is calculated by subtracting an individual raw score from the population mean and dividing this difference by the population SD.

### Correlation analysis

The spatial correlation of metabolite distributions was assessed using Pearson correlation coefficient (r_ij_), which was calculated according to the following equation:

$$r_{ij}=\frac{\text{cov}\left(I_{i}\left(r\right),I_{j}\left(r\right)\right)}{\sigma_{i}\sigma_{j}}$$

The cov represents for the covariance of the metabolite pairs, and the σ_i_ and σ_j_ signifies their SDs, respectively. The r_ij_ values were calculated through a home-designed program to obtain all r_ij_ values among the metabolites in a single run.

### Construction of the metabolite network

Metabolite remodeling network was constructed for each dataset group using all 550 identified metabolites. Only metabolite pairs with a correlation coefficient ≥0.5 were retained, and the DyNet algorithm (75) in Cytoscape 3.9.0 (https://cytoscape.org/download_old_versions.html) was used to analyze the remodeling of metabolite nodes in the sham/SNI group's constructed network.

### Statistical analysis

The results were visualized and analyzed using R-based packages or GraphPad Prism 6 (https://www.graphpad.com/support/prism-6-updates/). For the analysis of mechanical threshold changes over time, a repeated measures two-way ANOVA was conducted for multiple comparisons. Unpaired two-sided Student's *t* tests were employed to compare the differences between two groups, and *p* values were presented (*p* < 0.05 was considered statistically significant). Other specific statistical tests used for each dataset are detailed in the figure legends, including two-way ANOVA for the number of cells expressing c-Fos, unpaired *t* tests for the comparison of colabeled neurons proportions and electrophysiological characterization. For metabolite profiling by MS analysis, low-quality cells were filtered and excluded from downstream analyses as described. To ensure the integrity and objectivity of our findings, both the collection and analysis of data were conducted under blinded conditions. Specifically, experimenters were unaware of the group allocations throughout the data collection and analysis phases, thereby minimizing potential biases. All data collected from control and experimental groups in our experiments were analyzed blindly. Further details on data quantification and analysis for different experiments can be found above.

## Data availability

The data generated in this study are available from the corresponding author on reasonable request.

## Supporting information

This article contains supporting information.

## Conflict of interest

The authors declare that they have no conflicts of interest with the contents of this article.
