# Supplementary material for: Single-cell metabolomics profiling of somatosensory neurons in various stages of neuropathic pain
Source: J Biol Chem. 2025 Feb 13;301(3):108309. doi: 10.1016/j.jbc.2025.108309 (PMC11938157; doi:10.1016/j.jbc.2025.108309)
Supplement: Supporting information [file mmc1.docx]

**­­­­** Supplementary Information for

**Single-cell Metabolomics Profiling of Somatosensory Neurons in Various stages of Neuropathic Pain**

Lin Yi^1^, Tiepeng Liao^1,2^, Man Yuan^1^, Qi Chen^1^, Wei Xiong^1,2,3,4,^*, Hongying Zhu^1,2,3,4,^*

^1^ Hefei National Research Center for Physical Sciences at the Microscale, Division of Life Sciences and Medicine, University of Science and Technology of China, Hefei 230026, China

^2^ Anhui Province Key Laboratory of Biomedical Imaging and Intelligent Processing, Institute of Artificial Intelligence, Hefei Comprehensive National Science Center, Hefei 230088, China

^3^ CAS Key Laboratory of Brain Function and Disease, Hefei 230026, China

^4^ Anhui Province Key Laboratory of Biomedical Aging Research, Hefei 230026, China

*Correspondence: wxiong@ustc.edu.cn, zhuhy62@ustc.edu.cn

**Supplementary Figures**


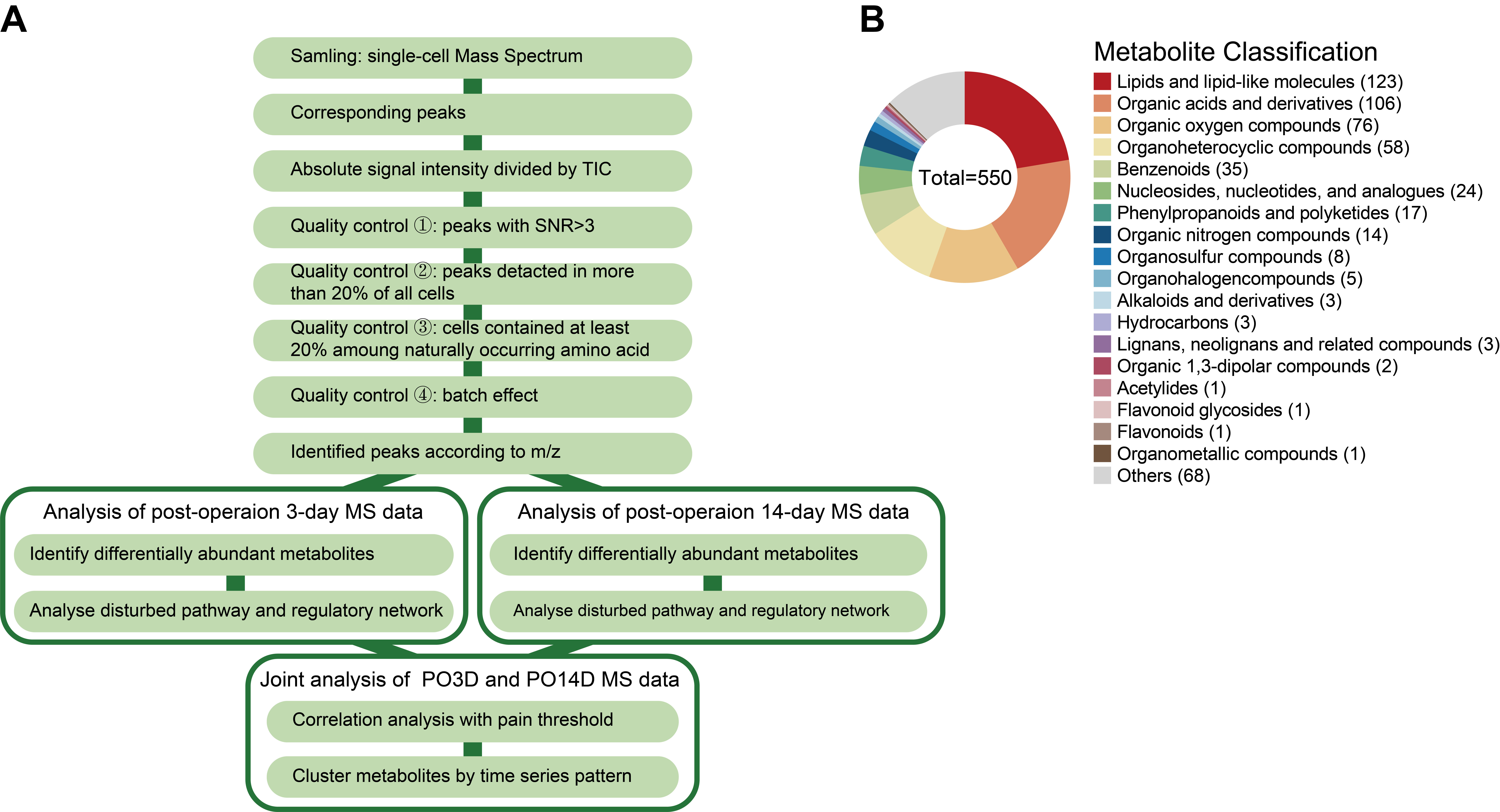


**Figure S1. Workflow for mass spectrometry metabolomic data processing and overview of metabolite detection and identification**

**(A)** Workflow of POD3 and POD14 mass spectrometry metabolomic data processing. **(B)** A pie chart depicting the sub-classification of all the metabolites that was identified after quality control screening.





**Figure S2. MS/MS spectra of several metabolites and their standards.**


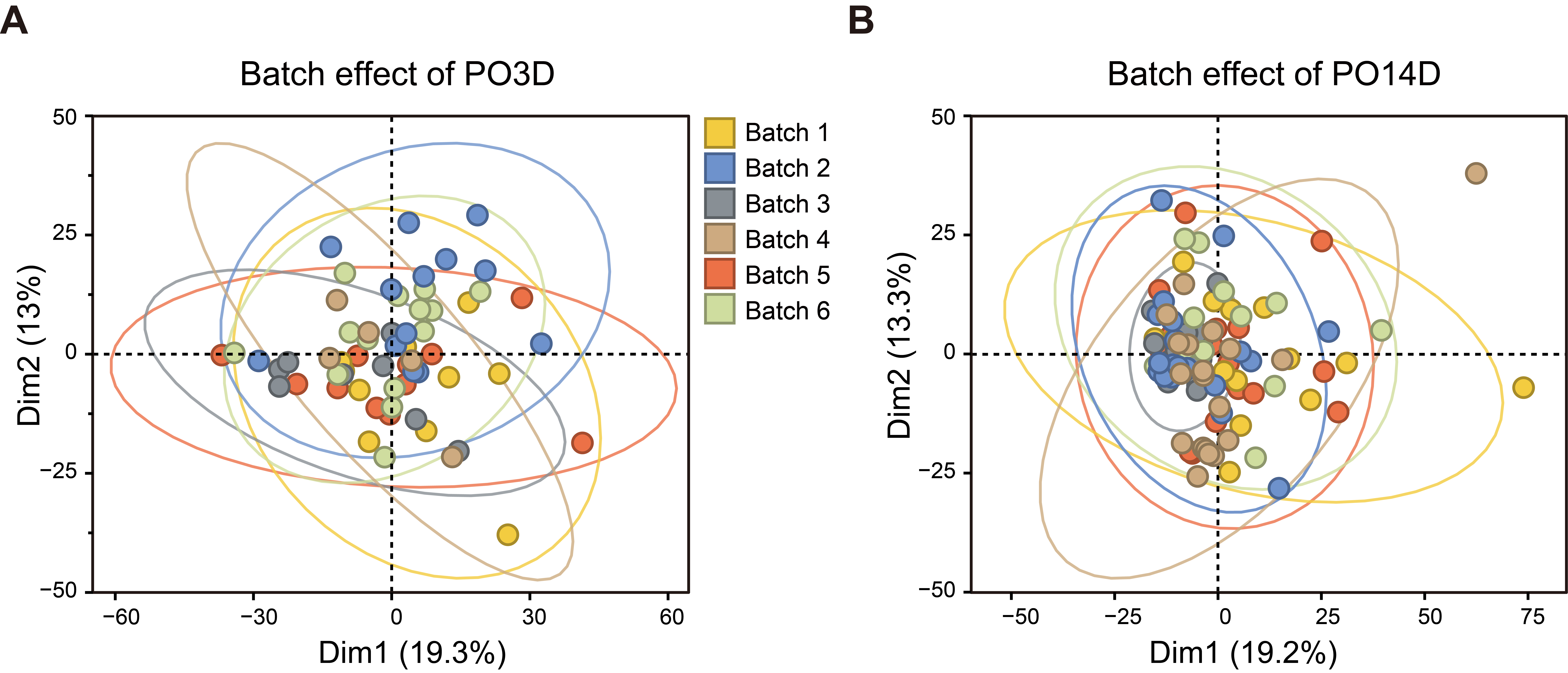


**Figure S3. Batch effect measured by PCA.**

**(A and B)** PCA analysis show no batch effect between 3 pairs of mice in **(A)** POD3 and **(B)** POD14 experiments respectively.


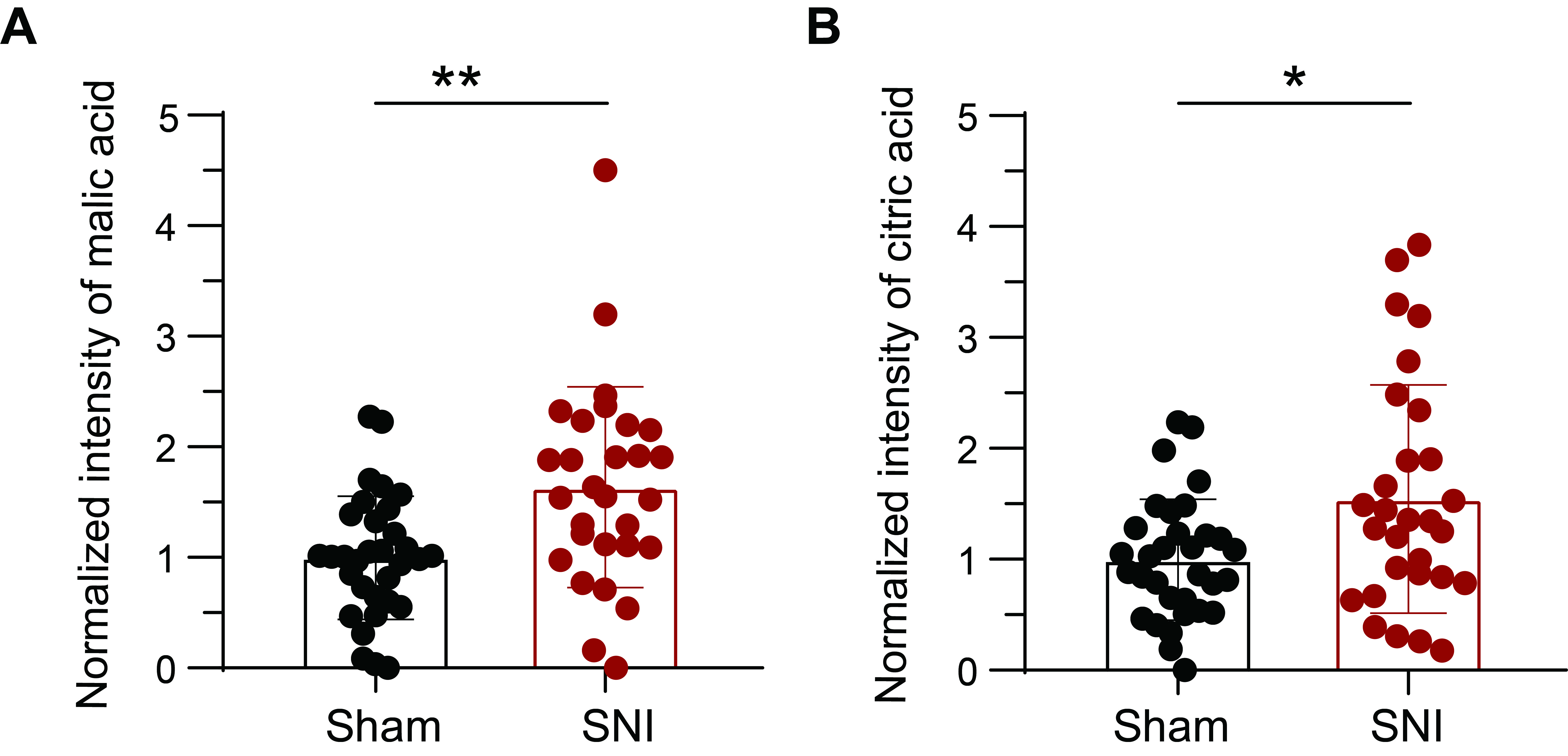


**Figure S4. Decreased levels of malic acid and citric acid in POD3 SNI mice compared to the sham mice.**

Data are presented as mean ± SD. * *p* <0.05, ** *p* <0.01 by unpaired t-tests; ns, not significant (*p* > 0.05).


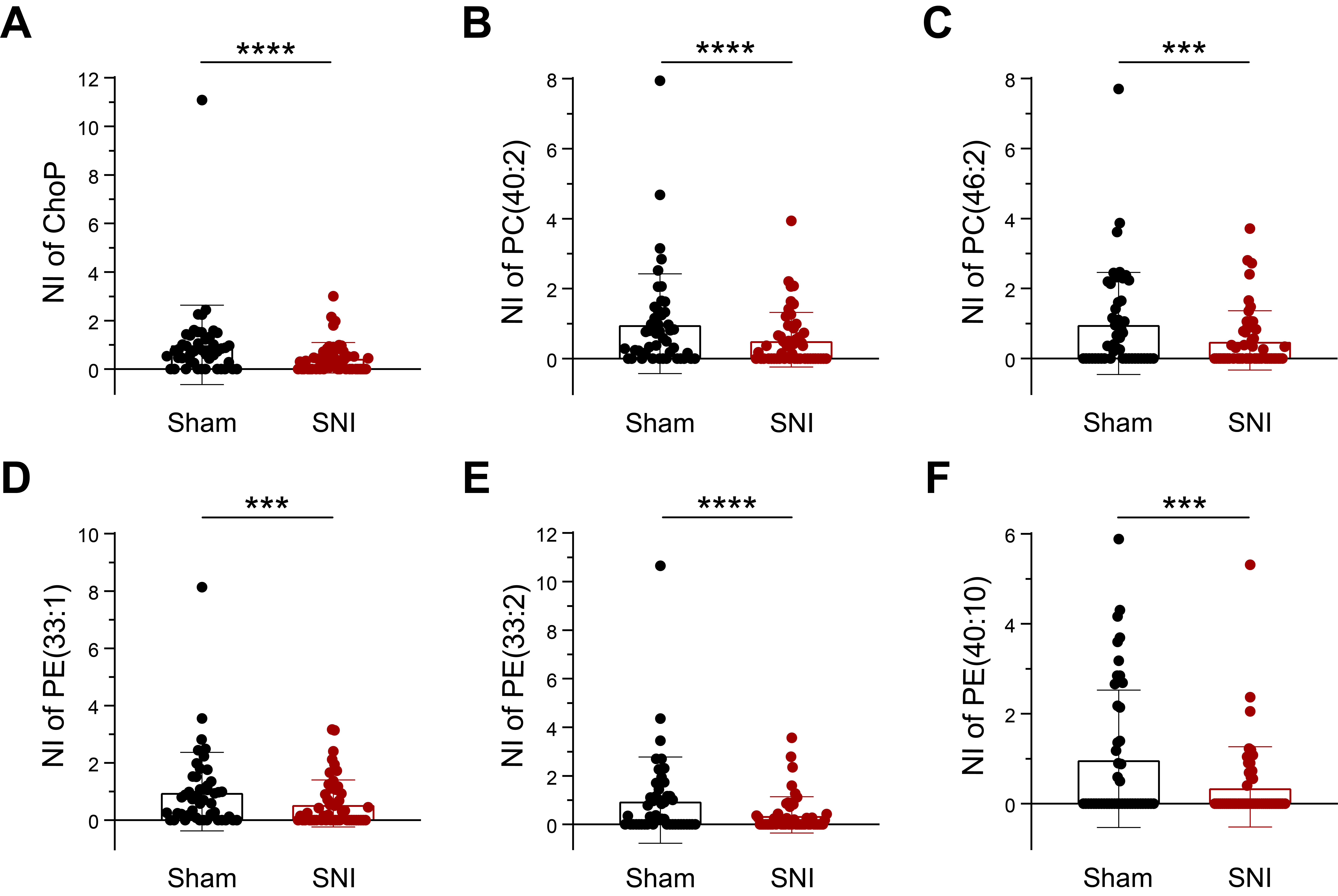


**Figure S5. Decreased levels of lipids in POD14 SNI mice compared to the sham mice.**

Data are presented as mean ± SD. **p* <0.05, ** *p* <0.01, *** *p* <0.001, **** *p* <0.0001 by unpaired t-tests; ns, not significant (*p* > 0.05).


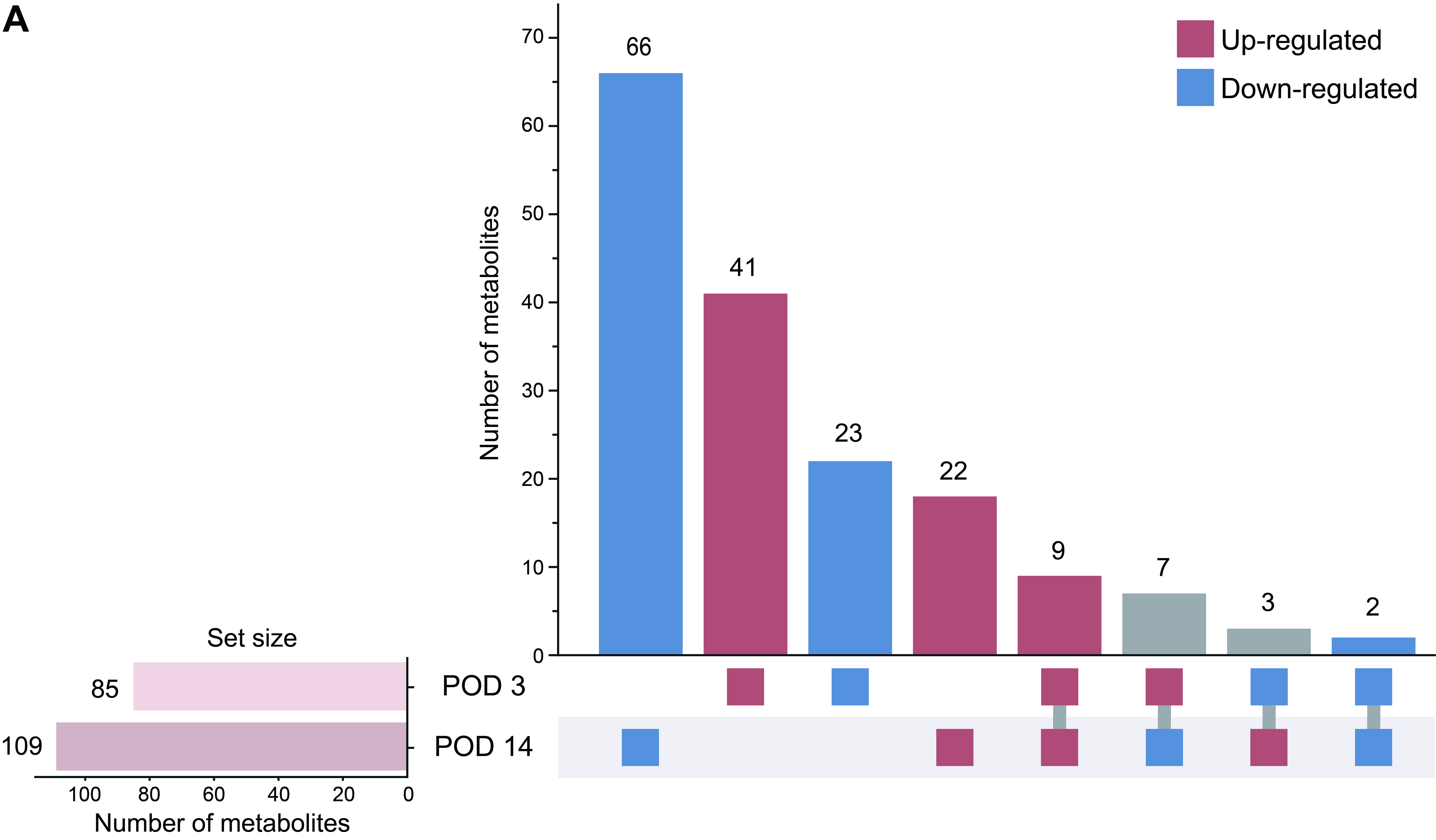


**Figure S6.** **Joint analysis of metabolic data at POD3 and POD14.**

**(A)** UpSet plot indicates the metabolites that are significantly dysregulated between SNI/sham groups on POD3 and POD14.

| **Table S1. Characteristic metabolites detected significantly regulated in POD3 SNI/sham mice** | | | | |
| --- | --- | --- | --- | --- |
| Name | Fold change | *p*-Value | HMDB ID  (Version 5.0) | Super Class |
| Glycine | 1.66 | 4.75E-03 | HMDB0000123 | Organic acids and derivatives |
| (Methylthio)-propanol | 2.00 | 2.89E-02 | HMDB0031716 | Organosulfur compounds |
| Alanine | 2.91 | 2.61E-02 | HMDB0000161 | Organic acids and derivatives |
| Alanine | 1.48 | 3.40E-02 | HMDB0000161 | Organic acids and derivatives |
| Serine | 3.46 | 3.19E-02 | HMDB0000187 | Organic acids and derivatives |
| Serine | 1.61 | 4.98E-03 | HMDB0000187 | Organic acids and derivatives |
| Pyroglutamic acid | 1.43 | 1.16E-02 | HMDB0000267 | Organic acids and derivatives |
| (Methylthio)methyl-butenal | 1.47 | 1.72E-02 | HMDB0031370 | Organic oxygen compounds |
| Imidazole-acetaldehyde | 10.25 | 2.49E-02 | HMDB0003905 | Organoheterocyclic compounds |
| Asparagine | 1.97 | 2.56E-03 | HMDB0000168 | Organic acids and derivatives |
| Adenine | 1.25 | 3.63E-02 | HMDB0000034 | Organoheterocyclic compounds |
| Succinic acid | 2.67 | 3.40E-02 | HMDB0000254 | Organic acids and derivatives |
| Trihydroxybenzene | 0.72 | 1.38E-02 | HMDB0013675 | Benzenoids |
| Lysine | 1.43 | 1.52E-02 | HMDB0000182 | Organic acids and derivatives |
| Hydroxylated lecithin | 9.15 | 2.85E-02 | HMDB0032332 | Organic acids and derivatives |
| Thiocyanatophenol | 0.49 | 3.51E-03 | HMDB0040578 | Organosulfur compounds |
| Pyrroline hydroxycarboxylic acid | 2.16 | 2.50E-03 | HMDB0001369 | Organic acids and derivatives |
| Guanine | 1.38 | 2.07E-02 | HMDB0000132 | Organoheterocyclic compounds |
| Ureidopropionic acid | 4.45 | 3.17E-03 | HMDB0000026 | Organic acids and derivatives |
| Histidine^*^ | 1.42 | 8.21E-03 | HMDB0000177 | Organic acids and derivatives |
| Malic acid | 1.64 | 2.07E-03 | HMDB0000156 | Organic acids and derivatives |
| Dimethyl-vinylthiazole | 1.89 | 4.88E-02 | HMDB0037285 | Organoheterocyclic compounds |
| Bromoacetaldehyde | 0.35 | 3.15E-02 | HMDB0060344 | Organohalogen compounds |
| Chloral hydrate | 1.63 | 5.04E-03 | HMDB0060451 | Organohalogen compounds |
| Phenylalanine | 1.36 | 7.86E-03 | HMDB0000159 | Organic acids and derivatives |
| Deoxy-glucitol | 2.67 | 4.21E-02 | HMDB0041500 | Organic oxygen compounds |
| Homogentisic acid | 0.37 | 1.81E-02 | HMDB0000130 | Benzenoids |
| Acetylserine | 1.52 | 4.62E-02 | HMDB0003011 | Organic acids and derivatives |
| Sulfinoalanine | 2.17 | 2.73E-02 | HMDB0000996 | Organic acids and derivatives |
| N-Acetyl-aspartic acid | 1.96 | 2.92E-02 | HMDB0000812 | Organic acids and derivatives |
| Citrulline | 1.45 | 3.28E-02 | HMDB0000904 | Organic acids and derivatives |
| Methylthio (propanoyloxy)propanoate | 2.24 | 4.70E-02 | HMDB0040003 | Organic acids and derivatives |
| Tyrosine | 1.38 | 1.67E-03 | HMDB0000158 | Organic acids and derivatives |
| Dihydroxy-nitrophenol | 2.58 | 1.27E-02 | HMDB0006200 | Benzenoids |
| N-Formyl-glutamic acid | 2.39 | 7.60E-03 | HMDB0003470 | Organic acids and derivatives |
| Carnitine | 2.12 | 1.42E-02 | HMDB0000062 | Organic nitrogen compounds |
| Pyridoxic acid | 0.46 | 4.16E-02 | HMDB0000017 | Organoheterocyclic compounds |
| Tryptophan | 7.33 | 1.91E-02 | HMDB0000929 | Organoheterocyclic compounds |
| Citric acid | 1.55 | 1.44E-02 | HMDB0000094 | Organic acids and derivatives |
| Cer(d16:1/20:3-2OH) | 0.66 | 4.20E-02 | HMDB0289858 | Organic oxygen compounds |
| Citric acid | 1.64 | 3.61E-02 | HMDB0000094 | Organic acids and derivatives |
| Cer(d18:1/24:1) | 1.63 | 4.46E-02 | HMDB0004953 | Lipids and lipid-like  molecules |
| Hydroxy-methylpyridine-dicarboxylate | 3.62 | 1.87E-02 | HMDB0006955 | Organoheterocyclic compounds |
| CE(18:3) | 0.56 | 3.04E-02 | HMDB0010370 | Lipids and lipid-like  molecules |
| SM(d16:1/16:0) | 0.57 | 2.91E-02 | HMDB0240616 | Lipids and lipid-like  molecules |
| DG(40:7) | 0.38 | 9.96E-03 | HMDB0056294 | Lipids and lipid-like  molecules |
| Cer(d17:1/20:5-3OH) | 0.65 | 3.54E-02 | HMDB0289924 |  |
| Cer(d17:1/PGE2) | 0.63 | 3.97E-02 | HMDB0289901 |  |
| Tyrosol 4-sulfate | 1.84 | 1.40E-03 | HMDB0041785 | Organic acids and derivatives |
| Stearic acid | 0.64 | 2.84E-02 | HMDB0000827 | Lipids and lipid-like  molecules |
| Chlorohippuric acid | 2.51 | 4.99E-02 | HMDB0001309 | Benzenoids |
| Hydroxy-N-formylkynurenine | 3.43 | 1.13E-02 | HMDB0004086 | Organic oxygen compounds |
| Methylselenopyruvate | 2.08 | 1.14E-03 | HMDB0060490 | Organic acids and derivatives |
| PE-NMe(36:3) | 1.70 | 2.91E-02 | HMDB0113341 | Lipids and lipid-like  molecules |
| PE(TXB2/16:0) | 2.10 | 1.11E-02 | HMDB0260921 |  |
| PG(22:5-O/i-14:0) | 0.48 | 4.09E-02 | HMDB0271343 |  |
| PGP(20:4-OH/i-12:0) | 3.20 | 4.75E-02 | HMDB0274808 |  |
| Sulfaquinoxaline | 0.35 | 4.13E-02 | HMDB0033139 | Organoheterocyclic compounds |
| PE(22:6-2OH/22:4) | 1.41 | 3.55E-02 | HMDB0284111 |  |
| Phosphopantothenate | 0.74 | 3.32E-02 | HMDB0001016 | Organic acids and derivatives |
| Dimethyl fukiic acid | 0.48 | 1.51E-02 | HMDB0029497 | Benzenoids |
| Cer(d18:1/23:0) | 2.11 | 4.79E-03 | HMDB0000950 | Lipids and lipid-like  molecules |
| Methylthioadenosine | 0.34 | 1.16E-02 | HMDB0001173 | Nucleosides, nucleotides, and analogues |
| Phosphatidylinositol-trisphosphate | 0.29 | 2.06E-02 | HMDB0004249 | Organic oxygen compounds |
| DG(18:1-O/0:0/i-19:0) | 2.49 | 7.60E-03 | HMDB0299882 |  |
| Cer(d18:0/24:1) | 0.42 | 1.12E-02 | HMDB0011769 | Lipids and lipid-like  molecules |
| Methylnonanedioyl-CoA | 0.47 | 3.61E-02 | HMDB0300597 |  |
| Adenosine monophosphate | 2.84 | 4.73E-02 | HMDB0000045 | Nucleosides, nucleotides, and analogues |
| TG(i-19:0/17:0/8:0) | 0.60 | 4.69E-02 | HMDB0110379 | Lipids and lipid-like  molecules |
| PC(O-18:1(9Z)/18:0) | 0.49 | 2.90E-02 | HMDB0013427 | Lipids and lipid-like  molecules |
| PA(i-24:0/i-16:0) | 0.61 | 3.70E-02 | HMDB0115942 | Lipids and lipid-like  molecules |
| PS(18:1-2OH/15:0) | 3.74 | 1.89E-02 | HMDB0281061 |  |
| Methoxy-hydroxy-nonaprenylbenzoate | 0.59 | 3.32E-02 | HMDB0304137 | Lipids and lipid-like  molecules |
| PE(40:10) | 3.59 | 1.55E-03 | HMDB0009690 | Lipids and lipid-like  molecules |
| PS(33:0) | 8.54 | 2.90E-02 | HMDB0112322 | Lipids and lipid-like  molecules |
| PS(36:2) | 0.64 | 4.26E-02 | HMDB0012380 | Lipids and lipid-like  molecules |
| PE(38:4) | 0.53 | 1.39E-02 | HMDB0011448 | Lipids and lipid-like  molecules |
| PC(34:2) | 0.45 | 3.28E-02 | HMDB0008101 | Lipids and lipid-like  molecules |
| PE(18:3-OH/DiMe) | 0.45 | 3.86E-02 | HMDB0285053 |  |
| SM(d20:1/20:3-2OH) | 2.30 | 3.08E-02 | HMDB0290733 |  |
| Desacetylcefotaxime | 2.33 | 3.73E-02 | HMDB0041868 | Organoheterocyclic compounds |
| phosphopantothenoyl-cysteine | 2.55 | 2.02E-02 | HMDB0304473 | Organic acids and derivatives |
| Gluconasturtiin | +∞ | 2.25E-02 | HMDB0038423 | Organic oxygen compounds |
| CL(i-16:0/i-17:0/i-17:0/i-17:0) | 2.01 | 4.94E-02 | HMDB0240019 |  |
| Melissic acid A | 3.23 | 4.40E-02 | HMDB0030925 | Lipids and lipid-like  molecules |
| Luteolin glucuronide | 3.40 | 5.67E-03 | HMDB0240541 | Phenylpropanoids and  polyketides |
| FAHFA(16:0/13-O-18:0) | 4.10 | 1.13E-02 | HMDB0112121 | Lipids and lipid-like  molecules |
| Ganglioside GM1  (d18:0/18:0) | 3.58 | 3.44E-02 | HMDB0011878 | Lipids and lipid-like  molecules |

Note: All the fragments of metabolites matching with that of standards were marked with star (*). The p-values were calculated by unpaired t-tests.

**Table S2. Pathway analysis of significantly regulated metabolites in POD3 data**

| Upregulated pathways | | | | |
| --- | --- | --- | --- | --- |
|  | Total | Hits | Raw *p* | Impact |
| Aminoacyl-tRNA biosynthesis^*^ | 48 | 9 | 4.78E-08 | 0.17 |
| Alanine, aspartate and glutamate metabolism^*^ | 28 | 5 | 9.32E-05 | 0.09 |
| Phenylalanine, tyrosine and tryptophan biosynthesis^*^ | 4 | 2 | 1.82E-03 | 1.00 |
| Glyoxylate and dicarboxylate metabolism^*^ | 32 | 4 | 2.09E-03 | 0.18 |
| Citrate cycle (TCA cycle) ^*^ | 20 | 3 | 4.78E-03 | 0.17 |
| Phenylalanine metabolism^*^ | 12 | 2 | 1.83E-02 | 0.36 |
| Histidine metabolism^*^ | 16 | 2 | 3.18E-02 | 0.27 |
| Selenocompound metabolism^*^ | 20 | 2 | 4.82E-02 | 0.00 |
| beta-Alanine metabolism | 21 | 2 | 5.27E-02 | 0.10 |
| Sphingolipid metabolism | 21 | 2 | 5.27E-02 | 0.27 |
| Glutathione metabolism | 28 | 2 | 8.79E-02 | 0.10 |
| Purine metabolism | 66 | 3 | 1.11E-01 | 0.08 |
| Cysteine and methionine metabolism | 33 | 2 | 1.16E-01 | 0.04 |
| Glycine, serine and threonine metabolism | 34 | 2 | 1.22E-01 | 0.48 |
| Taurine and hypotaurine metabolism | 8 | 1 | 1.35E-01 | 0.29 |
| Ubiquinone and other terpenoid-quinone biosynthesis | 9 | 1 | 1.51E-01 | 0.00 |
| Tryptophan metabolism | 41 | 2 | 1.66E-01 | 0.18 |
| Biotin metabolism | 10 | 1 | 1.66E-01 | 0.00 |
| Glycosylphosphatidylinositol (GPI)-anchor biosynthesis | 14 | 1 | 2.25E-01 | 0.00 |
| Arginine biosynthesis | 14 | 1 | 2.25E-01 | 0.23 |
| Butanoate metabolism | 15 | 1 | 2.39E-01 | 0.00 |
| Pantothenate and CoA biosynthesis | 19 | 1 | 2.92E-01 | 0.03 |
| Pyruvate metabolism | 22 | 1 | 3.30E-01 | 0.03 |
| Propanoate metabolism | 23 | 1 | 3.42E-01 | 0.00 |
| Lysine degradation | 25 | 1 | 3.66E-01 | 0.00 |
| Porphyrin and chlorophyll metabolism | 30 | 1 | 4.22E-01 | 0.00 |
| Glycerophospholipid metabolism | 36 | 1 | 4.83E-01 | 0.10 |
| Arginine and proline metabolism | 38 | 1 | 5.01E-01 | 0.05 |
| Pyrimidine metabolism | 39 | 1 | 5.11E-01 | 0.01 |
| Tyrosine metabolism | 42 | 1 | 5.37E-01 | 0.14 |
| Primary bile acid biosynthesis | 46 | 1 | 5.70E-01 | 0.02 |
| Metabolism of xenobiotics by cytochrome P450 | 64 | 1 | 6.94E-01 | 0.00 |
| Downregulated pathways | | | | |
|  | Total | Hits | Raw *p* | Impact |
| Linoleic acid metabolism^*^ | 5 | 1 | 2.96E-02 | 0.00 |
| Ubiquinone and other terpenoid-quinone biosynthesis | 9 | 1 | 5.27E-02 | 0.00 |
| Vitamin B6 metabolism | 9 | 1 | 5.27E-02 | 0.00 |
| alpha-Linolenic acid metabolism | 13 | 1 | 7.53E-02 | 0.00 |
| Pantothenate and CoA biosynthesis | 19 | 1 | 1.08E-01 | 0.02 |
| Phosphatidylinositol signaling system | 28 | 1 | 1.56E-01 | 0.01 |
| Inositol phosphate metabolism | 30 | 1 | 1.66E-01 | 0.01 |
| Cysteine and methionine metabolism | 33 | 1 | 1.81E-01 | 0.02 |
| Arachidonic acid metabolism | 36 | 1 | 1.96E-01 | 0.00 |
| Biosynthesis of unsaturated fatty acids | 36 | 1 | 1.96E-01 | 0.00 |
| Glycerophospholipid metabolism | 36 | 1 | 1.96E-01 | 0.09 |
| Steroid biosynthesis | 42 | 1 | 2.25E-01 | 0.00 |
| Tyrosine metabolism | 42 | 1 | 2.25E-01 | 0.06 |
| Metabolism of xenobiotics by cytochrome P450 | 64 | 1 | 3.24E-01 | 0.01 |
| Note: Metabolic pathways marked with an asterisk (*) indicate *p* < 0.05. | | | | |

**Table S3. Dynamic analysis of metabolic correlation networks in POD3 data**

| Name | DyNet Rewiring (Dn-score) | Dn-Score  (degree corrected) | Edge Count | HMDB ID |
| --- | --- | --- | --- | --- |
| Citrulline | 132.5 | 0.4538 | 292 | HMDB0000904 |
| Pyridoxic acid | 124.5 | 0.4770 | 261 | HMDB0000017 |
| Aminocyclopropanecarboxylic acid | 122 | 0.4207 | 290 | HMDB0036458 |
| Spermine | 121 | 0.4353 | 278 | HMDB0001256 |
| Aminomuconic acid semialdehyde | 117.5 | 0.4152 | 283 | HMDB0001280 |
| Asparagine | 117 | 0.4517 | 259 | HMDB0000168 |
| Methyl-methylthio-butene | 116 | 0.4113 | 282 | HMDB0032411 |
| Malic acid | 113.5 | 0.4127 | 275 | HMDB0000156 |
| Pamidronate | 112.5 | 0.4377 | 257 | HMDB0014427 |
| Sulfaquinoxaline | 112.5 | 0.4611 | 244 | HMDB0033139 |
| Acetylserine | 111.5 | 0.4685 | 238 | HMDB0003011 |
| Pyroglutamic acid | 110.5 | 0.4742 | 261 | HMDB0000267 |
| Glutaconic acid | 110.5 | 0.4234 | 233 | HMDB0000620 |
| Rhein | 108.5 | 0.4540 | 239 | HMDB0032876 |
| Tritriacontane | 108.5 | 0.4887 | 222 | HMDB0301832 |
| Glutamic acid | 107.5 | 0.3967 | 271 | HMDB0000148 |
| Pyrroline-hydroxy-carboxylate | 105.5 | 0.4953 | 213 | HMDB0002234 |
| PG(LTE4/i-13:0) | 105 | 0.4646 | 226 | HMDB0271188 |
| Trihydroxybenzene | 104 | 0.4228 | 233 | HMDB0013674 |
| PE(22:6-2OH/22:4) | 104 | 0.4464 | 246 | HMDB0284111 |
| Pyrrolidine | 103.5 | 0.4140 | 243 | HMDB0031641 |
| Amino-methyl-5H-pyrido[4,3-b]indole | 103.5 | 0.4259 | 250 | HMDB0033188 |
| Garcinia acid | 103 | 0.4813 | 214 | HMDB0031159 |
| TG(61:4) | 102 | 0.4016 | 254 | HMDB0043154 |
| Phosphatidylinositol trisphosphate | 100 | 0.4505 | 222 | HMDB0004249 |
| N-Acetyl-tyrosine | 99.5 | 0.3755 | 265 | HMDB0000866 |
| Anilazine | 99.5 | 0.4146 | 240 | HMDB0248437 |
| Aconitic acid | 98.5 | 0.3863 | 255 | HMDB0000072 |
| PI(20:4-2OH)/22:2) | 98.5 | 0.3988 | 247 | HMDB0277766 |
| Phenylpyruvic acid | 98 | 0.4100 | 239 | HMDB0000205 |
| Histidine | 97 | 0.4292 | 226 | HMDB0000177 |
| (methylthio)Methyl-butenal | 96.5 | 0.4232 | 228 | HMDB0031370 |
| Proline | 96 | 0.4085 | 235 | HMDB0000162 |
| Guanine | 96 | 0.4638 | 207 | HMDB0000132 |
| Isosorbide Mononitrate | 94.5 | 0.3826 | 247 | HMDB0015155 |
| Phosphorylcholine | 94.5 | 0.4219 | 224 | HMDB0001565 |
| Ethyl (acetylthio)butyrate | 94 | 0.4896 | 192 | HMDB0032259 |
| Cer(d18:1/24:1) | 94 | 0.3629 | 259 | HMDB0004953 |
| Gallate 3-sulfate | 94 | 0.4541 | 207 | HMDB0240519 |
| Mesylate | 93.5 | 0.3770 | 248 | HMDB0240280 |

Note: All the metabolites were ranked by DyNet rewiring score.

**Table S4. Characteristic metabolites detected significantly regulated in POD14 SNI/sham mice**

| Name | Fold change | *p*-Value | HMDB ID  ( Version 5.0) | Super Class |
| --- | --- | --- | --- | --- |
| Epinephrine | 3.64 | 3.38E-03 | HMDB0000068 | Benzenoids |
| Methylarsonite | 0.53 | 4.93E-02 | HMDB0012259 | Organometallic compounds |
| Acetonitrile | 0.49 | 1.43E-04 | HMDB0061869 | Organic nitrogen compounds |
| Bis(furanylmethyl) sulfide | 0.35 | 2.03E-02 | HMDB0041503 | Organoheterocyclic compounds |
| Pyrrolidine | 1.95 | 1.03E-02 | HMDB0031641 | Organoheterocyclic compounds |
| Chlorobenzoic acid | 0.30 | 4.34E-02 | HMDB0001544 | Benzenoids |
| Acetone | 0.34 | 1.37E-02 | HMDB0001659 | Organic oxygen compounds |
| Furanone | 0.77 | 3.46E-02 | HMDB0032330 | Organoheterocyclic compounds |
| Alanine | 1.23 | 4.36E-02 | HMDB0000161 | Organic acids and derivatives |
| Lactic acid | 0.52 | 3.74E-02 | HMDB0000190 | Organic acids and derivatives |
| Quinic acid | 2.82 | 1.19E-02 | HMDB0003072 | Organic oxygen compounds |
| Imidazolone | 0.10 | 3.87E-02 | HMDB0004363 | Organic acids and derivatives |
| (Methylthio)-propanol | 1.55 | 3.31E-02 | HMDB0031716 | Organosulfur compounds |
| Dimethylurea | 0.58 | 4.88E-03 | HMDB0029198 | Organic acids and derivatives |
| Oxalic acid | 0.25 | 1.80E-02 | HMDB0002329 | Organic acids and derivatives |
| Creatinine | 1.42 | 7.59E-03 | HMDB0000562 | Organic acids and derivatives |
| Trifluoroacetic acid | 0.66 | 2.39E-02 | HMDB0014118 | Organic acids and derivatives |
| Glycerol | 24.06 | 1.48E-03 | HMDB0000131 | Organic oxygen compounds |
| Proline^*^ | 1.43 | 3.49E-04 | HMDB0000162 | Organic acids and derivatives |
| Dimethyl sulfone | 0.62 | 4.07E-02 | HMDB0004983 | Organosulfur compounds |
| Methyl-methylthio-butene | 1.48 | 3.63E-04 | HMDB0032411 | Organosulfur compounds |
| Guanidoacetic acid | 0.53 | 8.40E-03 | HMDB0000128 | Organic acids and derivatives |
| Mesylate | 0.30 | 3.09E-02 | HMDB0240280 | Organic acids and derivatives |
| Niacinamide | 1.61 | 2.57E-02 | HMDB0001406 | Organoheterocyclic compounds |
| Ketobutyric acid | 0.61 | 2.57E-02 | HMDB0000005 | Organic acids and derivatives |
| Isovaleraldehyde | 0.45 | 1.62E-02 | HMDB0006478 | Organic oxygen compounds |
| Pyroglutamic acid | 1.74 | 3.54E-06 | HMDB0000267 | Organic acids and derivatives |
| (Methylthio)methyl-butenal | 1.72 | 1.14E-05 | HMDB0031370 | Organic oxygen compounds |
| Aspartic acid | 1.82 | 1.78E-02 | HMDB0000191 | Organic acids and derivatives |
| Inosine | 2.00 | 3.33E-02 | HMDB0000195 | Nucleosides, nucleotides, and  analogues |
| Aminobenzoic acid | 1.67 | 2.36E-02 | HMDB0001123 | Benzenoids |
| Trihydroxybenzene | 1.57 | 1.23E-02 | HMDB0013675 | Benzenoids |
| Spermidine^*^ | 0.24 | 5.66E-03 | HMDB0001257 | Organic nitrogen compounds |
| Glutamine | 1.74 | 3.56E-03 | HMDB0000641 | Organic acids and derivatives |
| (Methylthio)methyl-butenal | 1.84 | 6.90E-03 | HMDB0031370 | Organic oxygen compounds |
| Mevalonic acid | 1.64 | 9.41E-04 | HMDB0000227 | Lipids and lipid-like molecules |
| Methionine | 1.51 | 9.75E-03 | HMDB0000696 | Organic acids and derivatives |
| Aspartic acid^*^ | 1.73 | 1.04E-02 | HMDB0000191 | Organic acids and derivatives |
| Thiocyanatophenol | 2.02 | 2.98E-02 | HMDB0040578 | Organosulfur compounds |
| Butyl-ethyloxazole | 2.20 | 4.26E-03 | HMDB0037862 | Organoheterocyclic compounds |
| Histidine^*^ | 1.41 | 8.83E-03 | HMDB0000177 | Organic acids and derivatives |
| Dimethyl-vinylthiazole | 2.08 | 3.64E-02 | HMDB0037285 | Organoheterocyclic compounds |
| Aminomuconic acid semialdehyde | 1.58 | 2.48E-02 | HMDB0001280 | Organic acids and derivatives |
| Acetylserine | 1.70 | 8.28E-03 | HMDB0003011 | Organic acids and derivatives |
| Phenylalanine | 1.31 | 5.66E-03 | HMDB0000159 | Organic acids and derivatives |
| Ribose | 1.91 | 1.89E-03 | HMDB0000283 | Organic oxygen compounds |
| Homogentisic acid | 2.43 | 3.42E-02 | HMDB0000130 | Benzenoids |
| Citrulline | 1.78 | 1.58E-02 | HMDB0000904 | Organic acids and derivatives |
| Ascorbic acid | 1.89 | 1.87E-02 | HMDB0000044 | Organoheterocyclic compounds |
| Gulonolactone | 0.50 | 2.97E-02 | HMDB0003466 | Organoheterocyclic compounds |
| Glucose | 0.65 | 3.67E-02 | HMDB0000122 | Organic oxygen compounds |
| Hydroxy-glutamic acid | 1.73 | 1.90E-02 | HMDB0002273 | Organic acids and derivatives |
| Tyrosine | 1.41 | 4.47E-03 | HMDB0000158 | Organic acids and derivatives |
| Selenocystine | 0.68 | 2.48E-02 | HMDB0004122 | Organic acids and derivatives |
| Ascorbic acid | 1.51 | 1.69E-02 | HMDB0000044 | Organoheterocyclic compounds |
| Hydroxy-methylguanine | 0.64 | 4.31E-02 | HMDB0006037 | Organoheterocyclic compounds |
| Tryptophan | 1.62 | 1.14E-02 | HMDB0000929 | Organoheterocyclic compounds |
| Phosphorylcholine | 0.47 | 4.32E-02 | HMDB0001565 | Organic nitrogen compounds |
| Cer(d16:1/20:3-2OH) | 0.32 | 1.10E-02 | HMDB0289858 | Organic oxygen compounds |
| Gluconic acid | 0.49 | 6.80E-03 | HMDB0000625 | Organic oxygen compounds |
| N-Acetyl-tyrosine | 0.61 | 1.15E-02 | HMDB0000866 | Organic acids and derivatives |
| Isoniazid pyruvate | 0.21 | 7.68E-03 | HMDB0060664 | Organoheterocyclic compounds |
| Tetrachloro-methoxybenzene | 0.61 | 2.07E-02 | HMDB0029627 | Benzenoids |
| Trichloro-methoxybenzene | 0.50 | 5.95E-03 | HMDB0029643 | Benzenoids |
| SM(d18:1/18:0) | 0.57 | 3.54E-02 | HMDB0001348 | Lipids and lipid-like molecules |
| PC(30:1) | 0.32 | 4.54E-02 | HMDB0013403 | Lipids and lipid-like molecules |
| PS(20:5-OH/14:1) | 0.57 | 3.69E-02 | HMDB0280969 |  |
| PS(30:0) | 0.56 | 4.06E-02 | HMDB0012352 | Lipids and lipid-like molecules |
| Methylselenopyruvate | 0.63 | 5.89E-03 | HMDB0060490 | Organic acids and derivatives |
| PE(33:1) | 0.53 | 4.36E-02 | HMDB0008894 | Lipids and lipid-like molecules |
| PC(18:3-OH/20:5) | 0.45 | 3.30E-02 | HMDB0287750 |  |
| PE-NMe2(42:1) | 0.23 | 3.31E-02 | HMDB0114636 | Lipids and lipid-like molecules |
| PS(18:1-O/20:5) | 0.20 | 2.52E-02 | HMDB0282832 | Lipids and lipid-like molecules |
| PE(PGD1/20:1) | 0.74 | 4.16E-02 | HMDB0284809 |  |
| PGP(36:6) | 0.58 | 1.61E-02 | HMDB0013583 | Lipids and lipid-like molecules |
| PC(9M5/9D5) | 0.63 | 2.04E-02 | HMDB0061466 | Lipids and lipid-like molecules |
| TG(15:0/18:2/O-18:0) | 0.64 | 1.35E-02 | HMDB0043423 | Lipids and lipid-like molecules |
| Sulfaquinoxaline | 0.32 | 2.38E-02 | HMDB0033139 | Organoheterocyclic compounds |
| Monoethylhexyl phthalic acid | 0.57 | 5.22E-03 | HMDB0013248 | Benzenoids |
| PGP(PGD1/a-13:0) | 0.63 | 1.62E-02 | HMDB0274361 |  |
| Rhein | 0.55 | 9.54E-03 | HMDB0032876 | Benzenoids |
| PE(PGD1/DiMe) | 0.57 | 3.80E-02 | HMDB0284953 |  |
| PC(40:2) | 0.48 | 2.33E-02 | HMDB0008792 | Lipids and lipid-like molecules |
| (Chloro-hydroxy-butynyl)-bithiophene | 0.00 | 3.29E-02 | HMDB0033269 | Organoheterocyclic compounds |
| Rhein | 0.29 | 4.47E-02 | HMDB0032876 | Benzenoids |
| TG(34:0) | 0.53 | 4.70E-02 | HMDB0107807 | Lipids and lipid-like molecules |
| Cer(d20:1/18:0) | 0.52 | 3.80E-02 | HMDB0240682 | Lipids and lipid-like molecules |
| PE(22:6-2OH/22:4) | 0.68 | 4.59E-02 | HMDB0284111 |  |
| PG(18:1-2OH/a-25:0) | 0.60 | 4.93E-02 | HMDB0271000 | Benzenoids |
| Cer(d18:1/23:0) | 0.45 | 5.09E-04 | HMDB0000950 | Lipids and lipid-like molecules |
| CDP-DG(18:1-O/i-12:0) | 0.29 | 3.07E-02 | HMDB0293318 |  |
| DG(38:3) | 0.56 | 4.87E-02 | HMDB0007650 | Lipids and lipid-like molecules |
| PGP(TXB2/a-25:0) | 0.59 | 4.77E-02 | HMDB0274720 |  |
| Ganglioside GA2 (d18:1/16:0) | 0.36 | 4.29E-02 | HMDB0004890 | Lipids and lipid-like molecules |
| Galabiosylceramide (d18:1/26:0) | 0.63 | 2.19E-02 | HMDB0004839 | Lipids and lipid-like molecules |
| DG(42:3) | 0.69 | 2.78E-02 | HMDB0007800 | Lipids and lipid-like molecules |
| PIP(PGF1alpha/22:3) | 0.50 | 2.72E-02 | HMDB0280380 |  |
| PC(34:5) | 0.45 | 4.53E-02 | HMDB0011214 | Lipids and lipid-like molecules |
| PE(33:2) | 0.34 | 2.03E-02 | HMDB0009087 | Lipids and lipid-like molecules |
| TG(66:3) | 0.59 | 4.46E-02 | HMDB0049371 | Lipids and lipid-like molecules |
| PE(36:2) | 0.40 | 5.56E-03 | HMDB0011439 | Lipids and lipid-like molecules |
| CDP-DG(18:1-2OH/22:6) | 0.39 | 3.98E-02 | HMDB0292690 |  |
| Torasemide | 0.58 | 4.33E-02 | HMDB0014359 | Organoheterocyclic compounds |
| PS(18:1-2OH/15:0) | 0.19 | 4.35E-03 | HMDB0281061 |  |
| PE(40:10) | 0.28 | 3.27E-03 | HMDB0009690 | Lipids and lipid-like molecules |
| SM(d20:1/20:3-2OH) | 0.25 | 3.97E-02 | HMDB0290733 |  |
| PE(42:3) | 0.33 | 3.69E-02 | HMDB0011462 | Lipids and lipid-like molecules |
| PC(46:2) | 0.51 | 4.91E-02 | HMDB0008809 | Lipids and lipid-like molecules |
| Petanin | 5.90 | 4.52E-02 | HMDB0038095 | Phenylpropanoids and  polyketides |
| PS(46:5) | 0.36 | 4.26E-02 | HMDB0112931 | Lipids and lipid-like molecules |
| SM(d19:0/LTE4) | 0.24 | 3.22E-02 | HMDB0290625 |  |
| CL(16:0/18:2/i-22:0/i-22:0) | 0.39 | 1.06E-02 | HMDB0238978 | Lipids and lipid-like molecules |
| FAHFA(16:0/13-O-18:0) | 0.41 | 3.74E-02 | HMDB0112121 | Lipids and lipid-like molecules |

Note: All the fragments of metabolites matching with that of standards were marked with star (*). The p-values were calculated by unpaired t-tests.

**Table S5. Pathway analysis of significantly regulated metabolites in POD14 data**

| Upregulated pathways | | | | |
| --- | --- | --- | --- | --- |
|  | Total | Hits | Raw *p* | Impact |
| Aminoacyl-tRNA biosynthesis^*^ | 48 | 9 | 2.02E-09 | 0 |
| Arginine biosynthesis^*^ | 14 | 3 | 6.65E-04 | 0.22843 |
| Phenylalanine, tyrosine and tryptophan biosynthesis^*^ | 4 | 2 | 9.90E-04 | 1 |
| Alanine, aspartate and glutamate metabolism^*^ | 28 | 3 | 5.31E-03 | 0.33734 |
| Ubiquinone and other terpenoid-quinone biosynthesis^*^ | 9 | 2 | 5.71E-03 | 0 |
| Phenylalanine metabolism^*^ | 12 | 2 | 1.02E-02 | 0.35714 |
| Tryptophan metabolism^*^ | 41 | 3 | 1.55E-02 | 0.15897 |
| Nicotinate and nicotinamide metabolism^*^ | 15 | 2 | 1.59E-02 | 0.1943 |
| Tyrosine metabolism^*^ | 42 | 3 | 1.65E-02 | 0.21613 |
| Histidine metabolism^*^ | 16 | 2 | 1.80E-02 | 0.22131 |
| beta-Alanine metabolism^*^ | 21 | 2 | 3.03E-02 | 0 |
| D-Glutamine and D-glutamate metabolism | 6 | 1 | 7.72E-02 | 0 |
| Nitrogen metabolism | 6 | 1 | 7.72E-02 | 0 |
| Glycerolipid metabolism | 16 | 1 | 1.93E-01 | 0.23676 |
| Terpenoid backbone biosynthesis | 18 | 1 | 2.15E-01 | 0.11429 |
| Purine metabolism | 66 | 2 | 2.17E-01 | 0.00249 |
| Pantothenate and CoA biosynthesis | 19 | 1 | 2.26E-01 | 0 |
| Selenocompound metabolism | 20 | 1 | 2.36E-01 | 0 |
| Pentose phosphate pathway | 22 | 1 | 2.56E-01 | 0 |
| Galactose metabolism | 27 | 1 | 3.05E-01 | 0 |
| Glutathione metabolism | 28 | 1 | 3.15E-01 | 0.00709 |
| Glyoxylate and dicarboxylate metabolism | 32 | 1 | 3.51E-01 | 0 |
| Cysteine and methionine metabolism | 33 | 1 | 3.60E-01 | 0.10446 |
| Arginine and proline metabolism | 38 | 1 | 4.02E-01 | 0.0778 |
| Pyrimidine metabolism | 39 | 1 | 4.10E-01 | 0 |
| Downregulated pathways | | | | |
|  | Total | Hits | Raw *p* | Impact |
| Glycerophospholipid metabolism^*^ | 36 | 3 | 3.05E-03 | 0.20832 |
| Sphingolipid metabolism^*^ | 21 | 2 | 1.32E-02 | 0.03854 |
| Glycine, serine and threonine metabolism^*^ | 34 | 2 | 3.30E-02 | 0.02408 |
| Arginine and proline metabolism^*^ | 38 | 2 | 4.06E-02 | 0.05747 |
| Linoleic acid metabolism^*^ | 5 | 1 | 4.25E-02 | 0 |
| Valine, leucine and isoleucine biosynthesis | 8 | 1 | 6.72E-02 | 0 |
| Ascorbate and aldarate metabolism | 10 | 1 | 8.33E-02 | 0.25 |
| alpha-Linolenic acid metabolism | 13 | 1 | 1.07E-01 | 0 |
| Glycosylphosphatidylinositol (GPI)-anchor biosynthesis | 14 | 1 | 1.15E-01 | 0.00399 |
| Selenocompound metabolism | 20 | 1 | 1.60E-01 | 0 |
| beta-Alanine metabolism | 21 | 1 | 1.67E-01 | 0 |
| Pyruvate metabolism | 22 | 1 | 1.75E-01 | 0 |
| Pentose phosphate pathway | 22 | 1 | 1.75E-01 | 0.04712 |
| Propanoate metabolism | 23 | 1 | 1.82E-01 | 0.04061 |
| Glycolysis / Gluconeogenesis | 26 | 1 | 2.03E-01 | 0 |
| Glutathione metabolism | 28 | 1 | 2.17E-01 | 0.00719 |
| Cysteine and methionine metabolism | 33 | 1 | 2.51E-01 | 0.05983 |
| Arachidonic acid metabolism | 36 | 1 | 2.71E-01 | 0 |
| Metabolism of xenobiotics by cytochrome P450 | 64 | 1 | 4.33E-01 | 0 |

Note: Metabolic pathways marked with an asterisk (*) indicate *p* < 0.05.

**Table S6. Dynamic analysis of metabolic correlation networks in POD14 data**

| Name | DyNet Rewiring (Dn-score) | Dn-Score (degree corrected) | Edge Count | HMDB ID |
| --- | --- | --- | --- | --- |
| Trihydroxybenzene | 106.5 | 0.477578 | 223 | HMDB0013674 |
| Acetone | 103.5 | 0.433054 | 239 | HMDB0001659 |
| Glycine | 103.5 | 0.404297 | 256 | HMDB0000123 |
| Acetonitrile | 101 | 0.392996 | 257 | HMDB0061869 |
| Glucose | 100.5 | 0.440789 | 228 | HMDB0000122 |
| Piperidine | 99 | 0.464789 | 213 | HMDB0034301 |
| Oxymorphone | 99 | 0.39759 | 249 | HMDB0015323 |
| Bromoacetaldehyde | 98.5 | 0.464623 | 212 | HMDB0060344 |
| Gemcitabine diphosphate | 98 | 0.413502 | 237 | HMDB0060639 |
| Hydroxy-glutamic acid | 97.5 | 0.425764 | 229 | HMDB0002273 |
| Taurine | 97 | 0.391129 | 248 | HMDB0000251 |
| Lactic acid | 96 | 0.468293 | 205 | HMDB0000190 |
| Hydroxyanthraquinone | 95.5 | 0.375984 | 254 | HMDB0243898 |
| Dichlorodifluoromethane | 94 | 0.427273 | 220 | HMDB0029570 |
| Dimethylurea | 92.5 | 0.400433 | 231 | HMDB0029198 |
| Creatine | 92.5 | 0.474359 | 195 | HMDB0000064 |
| Aspartic acid | 91.5 | 0.442029 | 207 | HMDB0000191 |
| TG(62:5) | 91.5 | 0.448529 | 204 | HMDB0044017 |
| Methionine | 91 | 0.415525 | 219 | HMDB0000696 |
| Hydroxyglutamate semialdehyde | 89.5 | 0.374477 | 239 | HMDB0006556 |
| Cer(d18:1/23:0) | 89.5 | 0.454315 | 197 | HMDB0000950 |
| Butyric acid | 89 | 0.42381 | 210 | HMDB0000039 |
| Bromoacetaldehyde | 89 | 0.475936 | 187 | HMDB0060344 |
| Ethylacrylic acid | 89 | 0.415888 | 214 | HMDB0001862 |
| Iminoaspartic acid | 89 | 0.39207 | 227 | HMDB0001131 |
| Caffeate O-sulfate | 89 | 0.37395 | 238 | HMDB0041706 |
| dIDP | 89 | 0.494444 | 180 | HMDB0003536 |
| PC(22:5-O/DiMe) | 88.5 | 0.383117 | 231 | HMDB0289096 |
| Glycine | 88 | 0.39819 | 221 | HMDB0000123 |
| Phenylalanine | 88 | 0.376068 | 234 | HMDB0000159 |
| Glycerophosphate | 88 | 0.458333 | 192 | HMDB0000126 |
| Pamidronate | 88 | 0.455959 | 193 | HMDB0014427 |
| Inosine | 87.5 | 0.420673 | 208 | HMDB0000195 |
| Guanine | 87.5 | 0.377155 | 232 | HMDB0000132 |
| dioxo-gulonate | 87 | 0.483333 | 180 | HMDB0304040 |
| Luteolin 7-  glucuronide | 87 | 0.432836 | 201 | HMDB0240541 |
| Ketoleucine | 86.5 | 0.448187 | 193 | HMDB0000695 |
| Ketobutyric acid | 86 | 0.365957 | 235 | HMDB0000005 |
| Monoethylhexyl phthalic acid | 86 | 0.380531 | 226 | HMDB0013248 |
| Trihydroxybenzene | 106.5 | 0.477578 | 223 | HMDB0013674 |

Note: All the metabolites were ranked by DyNet rewiring score.

**Table S7. Characteristic metabolites of each cluster with different change patterns**

| Cluster 1 | | | | | | |
| --- | --- | --- | --- | --- | --- | --- |
| Name | 3d_sham_FC | 3d_sham_p | 14d_sham_FC | 14d_sham_p | 14d_3d_FC | 14d_3d_p |
| (Methylthio)-propanol | 2.00 | 0.0289 | 1.55 | 0.0331 | 0.78 | 0.3207 |
| Pyroglutamic acid | 1.43 | 0.0116 | 1.74 | 0.0000 | 1.22 | 0.0954 |
| (Methylthio)methyl-butenal | 1.47 | 0.0172 | 1.72 | 0.0000 | 1.17 | 0.2401 |
| Histidine | 1.42 | 0.0082 | 1.41 | 0.0088 | 1.00 | 0.9865 |
| Dimethyl-vinylthiazole | 1.89 | 0.0488 | 2.08 | 0.0364 | 1.11 | 0.7230 |
| Phenylalanine | 1.36 | 0.0079 | 1.31 | 0.0057 | 0.97 | 0.7445 |
| Citrulline | 1.45 | 0.0328 | 1.78 | 0.0158 | 1.22 | 0.2329 |
| Tyrosine | 1.38 | 0.0017 | 1.41 | 0.0045 | 1.02 | 0.8104 |
| Cluster 2 | | | | | | |
| Name | 3d_sham_FC | 3d_sham_p | 14d_sham_FC | 14d_sham_p | 14d_3d_FC | 14d_3d_p |
| Epinephrine | 1.30 | 0.4107 | 3.64 | 0.0034 | 2.81 | 0.0097 |
| Pyrrolidine | 1.09 | 0.3436 | 1.95 | 0.0103 | 1.79 | 0.0105 |
| Quinic acid | 0.47 | 0.1780 | 2.82 | 0.0119 | 6.04 | 0.0009 |
| Glycerol | 1.15 | 0.2682 | 24.06 | 0.0015 | 20.85 | 0.0016 |
| Proline | 1.01 | 0.9451 | 1.43 | 0.0003 | 1.42 | 0.0004 |
| Methyl-methylthio-butene | 1.02 | 0.8890 | 1.48 | 0.0004 | 1.46 | 0.0005 |
| Niacinamide | 0.84 | 0.5014 | 1.61 | 0.0257 | 1.91 | 0.0041 |
| Aminobenzoic acid | 0.91 | 0.6740 | 1.67 | 0.0236 | 1.83 | 0.0085 |
| Mevalonic acid | 1.11 | 0.4599 | 1.64 | 0.0009 | 1.48 | 0.0048 |
| Butyl-ethyloxazole | 0.41 | 0.0790 | 2.20 | 0.0043 | 5.32 | 0.0000 |
| Petanin | 0.75 | 0.3873 | 5.90 | 0.0452 | 7.84 | 0.0297 |
| Cluster 3 | | | | | | |
| Name | 3d_sham_FC | 3d_sham_p | 14d_sham_FC | 14d_sham_p | 14d_3d_FC | 14d_3d_p |
| Tryptophan | 7.34 | 0.0191 | 1.62 | 0.0114 | 0.22 | 0.0307 |
| Methylselenopyruvate | 2.08 | 0.0011 | 0.63 | 0.0059 | 0.30 | 0.0000 |
| PE(22:6-2OH(10S,17)/22:4) | 1.41 | 0.0355 | 0.68 | 0.0459 | 0.48 | 0.0001 |
| Cer(d18:1/23:0) | 2.11 | 0.0048 | 0.45 | 0.0005 | 0.22 | 0.0000 |
| PE(36:3) | 3.74 | 0.0189 | 0.19 | 0.0043 | 0.05 | 0.0025 |
| PE(40:10) | 3.59 | 0.0015 | 0.28 | 0.0033 | 0.08 | 0.0000 |
| SM(d20:1/20:3-2OH) | 2.30 | 0.0308 | 0.25 | 0.0397 | 0.11 | 0.0003 |
| FAHFA(16:0/13-O-18:0) | 4.09 | 0.0113 | 0.41 | 0.0374 | 0.10 | 0.0022 |
| Cluster 4 | | | | | | |
| Name | 3d_sham_FC | 3d_sham_p | 14d_sham_FC | 14d_sham_p | 14d_3d_FC | 14d_3d_p |
| Sulfaquinoxaline | 0.35 | 0.0413 | 0.32 | 0.0238 | 0.90 | 0.8085 |
| Cluster 5 | | | | | | |
| Name | 3d_sham_FC | 3d_sham_p | 14d_sham_FC | 14d_sham_p | 14d_3d_FC | 14d_3d_p |
| Cer(d16:1/20:3-2OH) | 0.66 | 0.0420 | 0.32 | 0.0110 | 0.48 | 0.0230 |
| Cluster 6 | | | | | | |
| Name | 3d_sham_FC | 3d_sham_p | 14d_sham_FC | 14d_sham_p | 14d_3d_FC | 14d_3d_p |
| Methylarsonite | 0.91 | 0.6143 | 0.53 | 0.0493 | 0.59 | 0.0326 |
| m-Chlorobenzoate | 0.92 | 0.7427 | 0.30 | 0.0434 | 0.33 | 0.0028 |
| Imidazolone | 1.05 | 0.8929 | 0.10 | 0.0387 | 0.09 | 0.0021 |
| Oxalic acid | 1.14 | 0.6763 | 0.25 | 0.0180 | 0.22 | 0.0016 |
| Trifluoroacetic acid | 1.07 | 0.6354 | 0.66 | 0.0239 | 0.62 | 0.0015 |
| Dimethyl sulfone | 1.14 | 0.4183 | 0.62 | 0.0407 | 0.54 | 0.0012 |
| Mesylate | 1.06 | 0.7032 | 0.30 | 0.0309 | 0.29 | 0.0000 |
| Pyridoxamine phosphate | 0.98 | 0.9544 | 0.45 | 0.0162 | 0.46 | 0.0342 |
| Selenocystine | 1.04 | 0.7924 | 0.68 | 0.0248 | 0.65 | 0.0022 |
| Tetrachloro-methoxybenzene | 1.12 | 0.4211 | 0.61 | 0.0207 | 0.54 | 0.0003 |
| Trichloro-methoxybenzene | 1.07 | 0.6574 | 0.50 | 0.0060 | 0.47 | 0.0001 |
| SM(d18:1/18:0) | 1.13 | 0.4424 | 0.57 | 0.0354 | 0.51 | 0.0007 |
| PC(30:1) | 1.21 | 0.2572 | 0.32 | 0.0454 | 0.26 | 0.0000 |
| PS(20:5-OH/14:1) | 1.19 | 0.3358 | 0.57 | 0.0369 | 0.48 | 0.0010 |
| PS(30:0) | 1.21 | 0.2984 | 0.56 | 0.0406 | 0.47 | 0.0010 |
| PE(33:1) | 1.23 | 0.2884 | 0.53 | 0.0436 | 0.44 | 0.0012 |
| PC(18:3-OH/20:5) | 1.26 | 0.4925 | 0.45 | 0.0330 | 0.36 | 0.0283 |
| PS(18:1-O/20:5) | 1.02 | 0.9601 | 0.20 | 0.0252 | 0.20 | 0.0040 |
| PE(PGD1/20:1) | 1.18 | 0.2373 | 0.74 | 0.0416 | 0.62 | 0.0019 |
| PGP(36:6) | 1.17 | 0.2939 | 0.58 | 0.0161 | 0.50 | 0.0001 |
| PC(9M5/9D5) | 1.22 | 0.2406 | 0.63 | 0.0204 | 0.52 | 0.0005 |
| TG(15:0/18:2/O-18:0) | 1.18 | 0.2446 | 0.64 | 0.0135 | 0.54 | 0.0002 |
| Monoethylhexyl phthalic acid | 0.87 | 0.5881 | 0.57 | 0.0052 | 0.65 | 0.0404 |
| PGP(PGD1/a-13:0) | 1.19 | 0.2156 | 0.63 | 0.0162 | 0.53 | 0.0002 |
| PE(PGD1/DiMe(13,5)) | 1.18 | 0.2970 | 0.57 | 0.0380 | 0.49 | 0.0006 |
| PC(40:2) | 1.21 | 0.3096 | 0.48 | 0.0233 | 0.39 | 0.0003 |
| (Chloro-hydroxy-butynyl)-bithiophene | 0.82 | 0.7483 | 0.00 | 0.0329 | 0.00 | 0.0097 |
| TG(34:0) | 1.28 | 0.2518 | 0.53 | 0.0470 | 0.42 | 0.0020 |
| Cer(d20:1/18:0) | 1.25 | 0.3081 | 0.52 | 0.0380 | 0.42 | 0.0024 |
| PG(18:1-2OH/a-25:0) | 1.36 | 0.3067 | 0.60 | 0.0493 | 0.44 | 0.0189 |
| CDP-DG(18:1-O/i-12:0) | 1.89 | 0.2013 | 0.29 | 0.0307 | 0.16 | 0.0068 |
| DG(38:3) | 1.72 | 0.0524 | 0.56 | 0.0487 | 0.33 | 0.0011 |
| PGP(TXB2/a-25:0) | 1.23 | 0.1770 | 0.59 | 0.0477 | 0.48 | 0.0002 |
| Ganglioside GA2  (d18:1/16:0) | 1.57 | 0.1756 | 0.36 | 0.0429 | 0.23 | 0.0025 |
| Galabiosylceramide (d18:1/26:0) | 1.16 | 0.2640 | 0.63 | 0.0219 | 0.54 | 0.0002 |
| DG(42:3) | 1.26 | 0.2979 | 0.69 | 0.0278 | 0.55 | 0.0092 |
| PIP(PGF1alpha/22:3) | 1.17 | 0.3598 | 0.50 | 0.0272 | 0.43 | 0.0001 |
| PC(34:5) | 1.24 | 0.4379 | 0.45 | 0.0453 | 0.36 | 0.0062 |
| PE(33:2) | 1.44 | 0.2087 | 0.34 | 0.0203 | 0.24 | 0.0017 |
| TG(66:3) | 1.56 | 0.1620 | 0.59 | 0.0446 | 0.38 | 0.0101 |
| PE(36:2) | 1.65 | 0.0733 | 0.40 | 0.0056 | 0.24 | 0.0004 |
| CDP-DG(18:1-2OH/22:6) | 1.46 | 0.2871 | 0.39 | 0.0398 | 0.27 | 0.0084 |
| PE(42:3) | 1.63 | 0.1448 | 0.33 | 0.0369 | 0.20 | 0.0016 |
| Bis(dichlorophenoxy)butanedioic acid | 1.36 | 0.1051 | 0.53 | 0.0072 | 0.39 | 0.0001 |
| PC(46:2) | 1.38 | 0.1170 | 0.51 | 0.0491 | 0.37 | 0.0004 |
| PS(46:5) | 1.50 | 0.2541 | 0.36 | 0.0426 | 0.24 | 0.0054 |
| SM(d19:0/LTE4) | 1.59 | 0.2978 | 0.24 | 0.0322 | 0.15 | 0.0075 |
| Cluster 7 | | | | | | |
| Name | 3d_sham_FC | 3d_sham_p | 14d_sham_FC | 14d_sham_p | 14d_3d_FC | 14d_3d_p |
| Trihydroxybenzene | 0.72 | 0.0138 | 1.57 | 0.0123 | 2.17 | 0.0001 |
| Thiocyanatophenol | 0.49 | 0.0035 | 2.02 | 0.0298 | 4.09 | 0.0003 |
| Homogentisic acid | 0.37 | 0.0181 | 2.43 | 0.0342 | 6.66 | 0.0012 |

Note: Metabolic pathways marked with an asterisk (*) indicate *p* < 0.05.

**Table S8. Correlation analysis of post-operative pain threshold with relative concentrations of metabolites detected in neuronal cytoplasm in SNI/sham mice**

| Positive correlation | | | | Negative correlation | | | |
| --- | --- | --- | --- | --- | --- | --- | --- |
| Rank No. | Name | r | *p* | Rank No. | Name | r | *p* |
| 1 | Rhein | 0.834 | 0.020 | 1 | Methionine | -0.719 | 0.068 |
| 2 | Unknown | 0.831 | 0.020 | 2 | Garcinia acid | -0.663 | 0.105 |
| 3 | Sulfaquinoxaline | 0.823 | 0.023 | 3 | Mevalonic acid | -0.634 | 0.126 |
| 4 | Unknown | 0.777 | 0.040 | 4 | Deoxy-glucitol | -0.616 | 0.141 |
| 5 | Methylthioadenosine | 0.729 | 0.063 | 5 | Unknown | -0.593 | 0.161 |
| 6 | Unknown | 0.728 | 0.063 | 6 | Creatine | -0.587 | 0.166 |
| 7 | Phosphoribosyl pyrophosphate | 0.717 | 0.070 | 7 | xi-Dihydro-dimethylthiazole | -0.587 | 0.166 |
| 8 | Ganglioside GA2  (d18:1/22:0) | 0.711 | 0.073 | 8 | Ascorbic acid | -0.576 | 0.176 |
| 9 | Phosphatidylinositol-trisphosphate | 0.707 | 0.076 | 9 | Unknown | -0.553 | 0.198 |
| 10 | Damascenone | 0.696 | 0.083 | 10 | Succinylacetoacetate | -0.552 | 0.199 |
| 11 | PC(20:4-3OH/14:1) | 0.695 | 0.083 | 11 | N-Methylene-ethenamine | -0.541 | 0.210 |
| 12 | Unknown | 0.693 | 0.084 | 12 | Adenosine triphosphate (ATP) | -0.535 | 0.216 |
| 13 | Unknown | 0.671 | 0.099 | 13 | Valine | -0.527 | 0.225 |
| 14 | Feruloylquinic acid | 0.670 | 0.100 | 14 | Ascorbic acid | -0.517 | 0.235 |
| 15 | Spermidine | 0.633 | 0.127 | 15 | Proline | -0.512 | 0.241 |
| 16 | Isovaleraldehyde | 0.630 | 0.130 | 16 | Dimethyl-vinylthiazole | -0.506 | 0.247 |
| 17 | Phosphoribosyl pyrophosphate | 0.594 | 0.160 | 17 | Tyrosine | -0.505 | 0.247 |
| 18 | Selenocystine | 0.593 | 0.160 | 18 | Phenylalanine | -0.505 | 0.247 |
| 19 | N-Acetyl-tyrosine | 0.593 | 0.161 | 19 | Creatinine | -0.496 | 0.258 |
| 20 | Unknown | 0.590 | 0.163 | 20 | Methyl-butene-thiol | -0.484 | 0.271 |
| 21 | Rhein | 0.587 | 0.166 | 21 | Unknown | -0.480 | 0.275 |
| 22 | Unknown | 0.579 | 0.173 | 22 | Methyl-methylthio-butene | -0.477 | 0.279 |
| 23 | Unknown | 0.578 | 0.175 | 23 | Unknown | -0.468 | 0.289 |
| 24 | Isoniazid pyruvate | 0.573 | 0.178 | 24 | Inosine | -0.458 | 0.301 |
| 25 | CDP-DG(18:1-2OH/22:6) | 0.573 | 0.178 | 25 | Ethanolamine | -0.457 | 0.303 |
| 26 | Unknown | 0.570 | 0.182 | 26 | Unknown | -0.457 | 0.303 |
| 27 | Trimethyl-1H-pyrrole | 0.564 | 0.188 | 27 | Aspartic acid | -0.456 | 0.304 |
| 28 | Unknown | 0.563 | 0.188 | 28 | Niacinamide | -0.454 | 0.306 |
| 29 | Phosphoglyceric acid | 0.561 | 0.191 | 29 | Ascorbic acid | -0.451 | 0.310 |
| 30 | Cystine | 0.560 | 0.191 | 30 | glucose | -0.449 | 0.312 |
| 31 | Unknown | 0.554 | 0.197 | 31 | Quinic acid | -0.446 | 0.316 |
| 32 | Diacetyl | 0.553 | 0.198 | 32 | Glutamine | -0.444 | 0.318 |
| 33 | Phosphorylcholine | 0.544 | 0.206 | 33 | Unknown | -0.441 | 0.322 |
| 34 | Raphanusanin | 0.542 | 0.208 | 34 | Unknown | -0.437 | 0.327 |
| 35 | Methylnonanedioyl-CoA | 0.541 | 0.210 | 35 | Tryptophan | -0.434 | 0.331 |
| 36 | Unknown | 0.540 | 0.211 | 36 | Pyroglutamic acid | -0.428 | 0.338 |
| 37 | Pyridoxic acid | 0.536 | 0.215 | 37 | Butyl-ethyloxazole | -0.428 | 0.338 |
| 38 | Nicotinic acid | 0.531 | 0.220 | 38 | Choline | -0.426 | 0.340 |
| 39 | Cyclohexane | 0.531 | 0.220 | 39 | Arginylalanine | -0.426 | 0.341 |
| 40 | Unknown | 0.530 | 0.221 | 40 | Unknown | -0.424 | 0.343 |
| 41 | Cellulose, microcrystalline | 0.528 | 0.223 | 41 | Succinic acid | -0.419 | 0.349 |
| 42 | Unknown | 0.527 | 0.224 | 42 | Desacetylcefotaxime | -0.418 | 0.351 |
| 43 | Anilazine | 0.523 | 0.228 | 43 | Histidine | -0.415 | 0.355 |
| 44 | Octacosane | 0.517 | 0.235 | 44 | (Methylthio)methyl-butenal | -0.411 | 0.360 |
| 45 | Unknown | 0.515 | 0.237 | 45 | PGP(20:4-OH/i-12:0) | -0.408 | 0.364 |
| 46 | Glycine | 0.511 | 0.241 | 46 | Adenosine triphosphate (ATP) | -0.407 | 0.364 |
| 47 | Unknown | 0.508 | 0.245 | 47 | Glutamic acid | -0.404 | 0.369 |
| 48 | Unknown | 0.504 | 0.249 | 48 | Unknown | -0.403 | 0.370 |
| 49 | (Amino-carboxypropyl)uridine | 0.500 | 0.253 | 49 | Glycerol | -0.402 | 0.372 |
| 50 | Unknown | 0.497 | 0.256 | 50 | Unknown | -0.401 | 0.372 |

Note: r denotes the Pearson correlation coefficient, and *p* denotes the corresponding *p*-value.
